# Integrated multi-omics analysis of renal metabolism in domestic cats with spontaneous chronic kidney disease

**DOI:** 10.1038/s42003-025-09164-8

**Published:** 2025-12-13

**Authors:** Qinghong Li, Ornella Cominetti, James A. Holzwarth, Stacie Summers, Xu Wang, Loïc Dayon

**Affiliations:** 1 Nestlé Research Hub St. Louis, St. Louis, MO USA; 2 College of Veterinary Medicine, Auburn University, Auburn, AL USA; 3 Nestlé Institute of Food Safety and Analytical Sciences, Nestlé Research, Lausanne, Switzerland; 4 Carlson College of Veterinary Medicine, Oregon State University, Corvallis, OR USA; 5 Institut des Sciences et Ingénierie Chimiques, Ecole Polytechnique Fédérale de Lausanne (EPFL), Lausanne, Switzerland

**Keywords:** Transcriptomics, Proteomics, Chronic kidney disease, Metabolism, Kidney

## Abstract

Chronic kidney disease (CKD) is the leading cause of mortality in aged cats. After injury, feline kidneys undergo extensive metabolic reprogramming, but a comprehensive evaluation is lacking. Here we show a multi-omics study including serum metabolomics from 14 healthy control, 15 early stages, 6 late stages CKD cats, and renal cortical and medullary tissue RNA sequencing and proteomics. The analysis reveals a spatiotemporal pattern of gene and protein expression changes. In the early stages, there are 6 differentially expressed genes in the cortex, while nearly 2000 in the medulla. The number in the cortex increases to more than 4000 in late stages. The study provides evidence of deranged bioenergetics in CKD: circulating fatty acids and acylcarnitines accumulate, while genes and proteins involved in fatty acid transport and oxidation are downregulated. Glucose and pyruvate metabolism is altered. Impaired glutamine metabolism contributes to both energy deficiency and acid-base imbalance. Additionally, there is a downregulation of redox enzymes, and overexpression of proinflammatory mediators in CKD. Gene expression of *TGFβ1* is strongly and positively correlated with that of other fibrogenic genes. Finally, oxygen homeostasis is disrupted. Hypoxia signaling is upregulated, while expression of *SGLT2* gene and protein is downregulated in cats with CKD. The data unveil profound metabolic abnormalities and adaptations in feline CKD.

## Abstract (web summary)

Multiomic integration and analysis on systemic metabolome, renal tissue transcriptome and proteome reveal profound metabolic abnormalities and adaptations in bioenergetics, oxygen and redox homeostasis in feline chronic kidney disease.

## Introduction

Chronic kidney disease (CKD) is the most common cause of mortality in aged cats, affecting >30% of cats aged 10 years or older^1,2^. Despite differing etiology, feline CKD is similar to human CKD in terms of clinical pathology, disease diagnosis and progression^3,4^. Tubulointerstitial fibrosis, a common lesion in both species, is present in the early stages of feline CKD, but typically a hallmark of end-stage CKD in humans^5^. Diabetes is the most common cause of CKD in people^6^, but does not appear to be a significant risk factor for cats^7^. Naturally occurring CKD in cats is considered a model of renal tubulointerstitial fibrosis^8,9^. Research in feline CKD can provide insights into the mechanisms of CKD in humans, and vice versa.

The kidney is a metabolically active organ rich in mitochondria^10,11^. The proximal tubular cells (PTCs) in the cortex have a high oxygen consumption rate, which makes them particularly susceptible to hypoxic injury^12–14^. Different segments of the nephron utilize different energy substrates to meet their specific metabolic needs^12,15^. Under physiological conditions, PTCs can readily metabolize fatty acids, glutamine, and lactate to generate ATP via oxidative phosphorylation (OXPHOS), while other parts of the kidney such as glomeruli oxidize glucose by aerobic glycolysis and inner medulla by anaerobic glycolysis^15^. After injury, the kidney reprograms to shift its energy reliance, which increases its oxygen consumption, becoming more vulnerable to hypoxia and oxidative injury^16,17^. Hypoxia is thought to play a significant role in the development of CKD by initiating chronic kidney inflammation and tubular fibrosis in both humans^13^ and cats^18,19^. However, the understanding on renal adaptations in energy metabolism and the mechanisms driving hypoxia and fibrosis in CKD remains incomplete.

Advancements in deep sequencing and mass spectrometry (MS) represent significant progress in the field of “omics”, providing new opportunities to study kidney health and disease. For example, a proteome study identified key proteins differentially expressed in renal extracellular matrix in rodent models of CKD and improved the understanding of kidney fibrosis^20,21^. In cats with experimental ischemia-induced CKD, transcriptomic analyses on renal tissues showed upregulations of proinflammatory and profibrotic pathways^22^, and cats with naturally occurring CKD showed downregulation of tubular uremic transporters^23^. Here, integration of serum metabolome with renal tissue transcriptome and proteome in cats with different stages of CKD provides a detailed depiction of the spatiotemporal metabolic landscape of feline CKD. The study highlights multiple factors that may contribute to the development of CKD in cats.

## Results

**Fig. 1:**
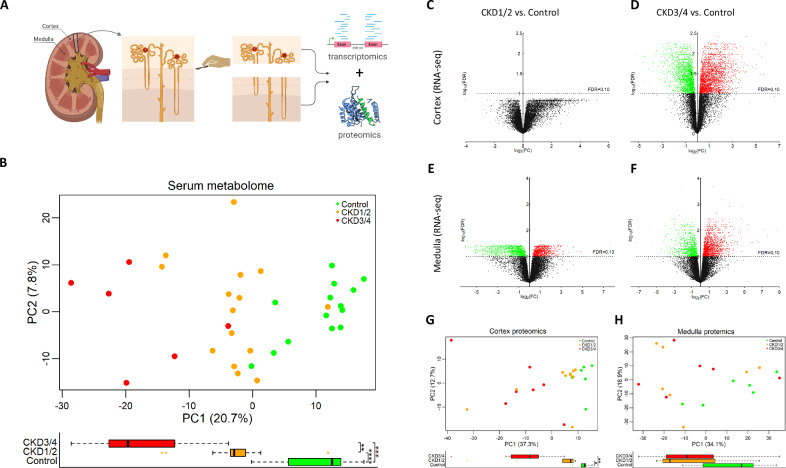
Study overview. **A** Renal sample procurement. Cortex and medulla tissues are separated for RNA sequencing (RNA-seq) and proteomic analysis. **B** PCA plot of serum metabolome (scatter plot, top panel). Separation is visible between the healthy control group, CKD1/2 group, and CKD3/4 group in PC1 (box plot, bottom panel). **C**–**F** Volcano plots of RNA-seq transcriptome from (**C**, **D**) renal cortex and (**E**, **F**) medulla tissues. Transcripts are arbitrarily colored to illustrate up- (red) and down-regulations (green) between (**C**, **E**) early stages of CKD (CKD1/2) versus control, and (**D**, **F**) late stages of CKD (CKD3/4) versus control. Spatiotemporal patterns of gene expression changes are evident. **G**, **H** PCA plot of renal cortex and medulla proteome, respectively. **B**, **G**, **H** The boxplots below the PCA plots show comparison in PC1 among the three groups. The whiskers extend to 1.5 times the interquartile range from the box. Sample size for metabolomics (**B**): control (*n* = 14), CKD1/2 (*n* = 15), and CKD3/4 (*n* = 6). Sample size for proteomics in the cortex (**G**): control (*n* = 6), CKD1/2 (*n* = 10), and CKD3/4 (*n* = 7); in the medulla (**H**): control (*n* = 6), CKD1/2 (*n* = 7), and CKD3/4 (*n* = 6). *P < 0.05, **P < 0.01, ***P < 0.001, ****P < 0.0001. Panel (**A**) was created in BioRender.com. PCA principal component (PC) analysis, CKD1/2 IRIS stage 1 and stage 2 chronic kidney disease, CKD3/4 IRIS stage 3 and stage 4 chronic kidney disease.

Serum samples were collected from 35 cats (cohort 1, Supplementary Table 1), and renal tissues were collected from 23 cats that were euthanized for humane reasons unrelated to the study (cohort 2, Supplementary Table 2). Cortex and medulla were separated, resulting in 21 cortical and 18 medullary samples for RNA sequencing (RNA-seq), and 23 cortex and 19 medulla samples for proteomic analyses (Fig. 1A). Sex was not different between CKD and healthy cats in either cohort. Age was different between groups in cohort 1, but not in cohort 2.

### Metabolome, transcriptome and proteome analyses

Serum untargeted metabolomic study identified 970 metabolites. The PCA analysis shows a global shift in metabolome profile (Fig. 1B, scatter plot). Both CKD groups are significantly different than the control group in PC1 (both *P* < 0.001, Fig. 1B, box plot). Linear regression analysis identifies 291 differential metabolites of known identities (all FDR < 0.10, Supplementary Data 1).

There are 14987 and 15074 transcripts that meet the minimum expression threshold in the cortex and medulla, respectively. Global transcriptome changes in the cortex and medulla in response to CKD are evident in PCA plots (Supplementary Fig. 1A, B, respectively) and heat maps (Supplementary Fig. 1C, D, respectively). The volcano plots reveal spatiotemporal patterns in global gene expression changes. In the cortex, there is a drastic increase in the number of differentially expressed genes (DEGs) from 6 in CKD1/2 to 4616 in CKD3/4 compared to control (Fig. 1C, D). In the medulla, there are 1995 DEGs in CKD1/2, and 2484 DEGs in CKD3/4 versus control (Fig. 1E, F), between which 879 (879/1995 = 44.1%) are in common.

Protein expression follows similar spatiotemporal patterns. In the cortex, there is no differentially expressed protein (DEP) identified in CKD1/2, but 1107 DEPs in CKD3/4 when compared with control. In the medulla, there is no DEP in CKD1/2, and 226 DEPs in CKD3/4. The PCA plot shows a clear separation between CKD groups and control group in the cortex, but not medulla (Fig. 1G, H).

No significant sex effect on the global profiles of metabolome, transcriptome, or proteome is observed (Supplementary Fig. 2A–C).

### Deranged renal energy metabolism in feline CKD

#### Impaired FA transport (FAT) and oxidation in mitochondria and peroxisomes

**Fig. 2:**
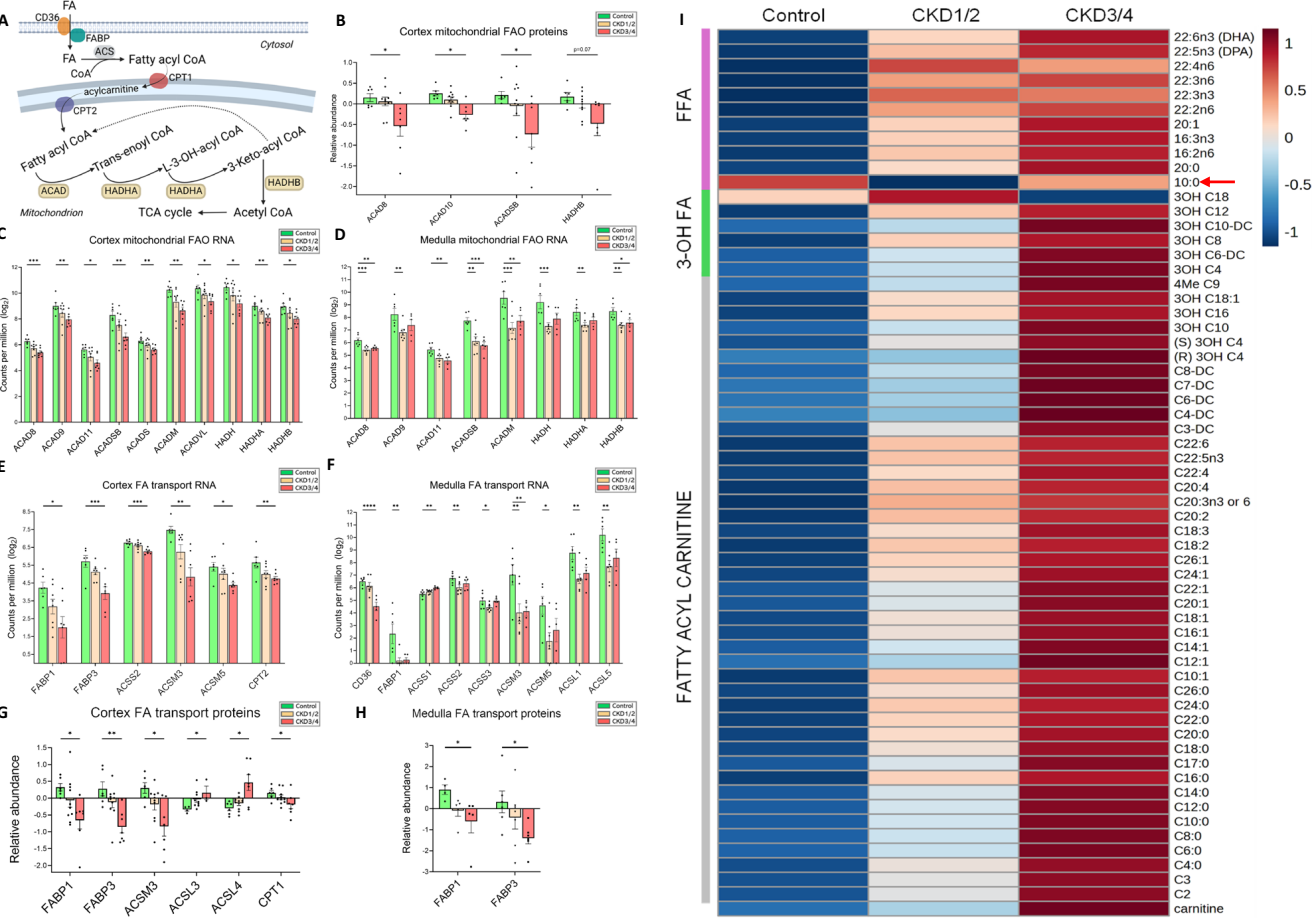
Aberrant FAO in feline CKD. **A** Schematic of FAT and FAO. Uptake of FAs into the cytoplasm through FAT proteins CD36 and FABPs, and transport into mitochondrial matrix through ACS and CPT-mediated carnitine shuttle pathway. The MTP complex is composed of 4 HADHA and 4 HADHB subunits and is specific for LCFA β-oxidation. In mitochondria, ACADs catalyze the first and rate-limiting step of FAO, while HADHA and HADHB are responsible for the rest of three reactions. **B** Expression of FAO enzyme proteins in the cortex. Negative values in protein expression are the results of log2 transformation on data <1. **C**, **D** Expression of FAO genes in the cortex and medulla, respectively. **E**, **F** Expression of FAT genes in the cortex and medulla, respectively. **G**, **H** Expression of FAT proteins in the cortex and medulla, respectively. **I** Heatmap is created using the mean concentrations of serum FFAs, 3-OH FAs, fatty acyl carnitines, and carnitine of the three groups: control, CKD1/2, and CKD3/4. Red arrow points to capric acid (C10:0). Color key for metabolite concentration: maroon (high) and blue (low). Sample size for RNA-seq experiments in the cortex (**C**, **E**): control (*n* = 6), CKD1/2 (*n* = 8), CKD3/4 (*n* = 7); in the medulla (**D**, **F**): control (*n* = 6), CKD1/2 (*n* = 7), CKD3/4 (*n* = 5); Sample size for proteomics experiments in the cortex (**B**, **G**): control (*n* = 6), CKD1/2 (*n* = 10), and CKD3/4 (*n* = 7) and in the medulla (**H**): control (*n* = 6), CKD1/2 (*n* = 7), and CKD3/4 (*n* = 6). Error bars represent the SEM. *P < 0.05, **P < 0.01, ***P < 0.001, ****P < 0.0001. Panel A was created in BioRender.com. FA fatty acid, LCFA long-chain fatty acid, FAT FA transport, FAO FA oxidation, CD36 FA translocase, FABP FA binding protein, ACS acyl-CoA synthetase, CPT carnitine palmitoyltransferase, MTP mitochondrial trifunctional protein, HADHA MTP alpha subunit, HADHB MTP beta subunit, ACAD acyl-CoA dehydrogenases, FFA free fatty acid, 3-OH FA 3-hydroxy fatty acid.

Long-chain FA (LCFA) β-oxidation (FAO) generates acetyl-CoA, which enters the tricarboxylic acid (TCA) cycle to produce ATP via OXPHOS (Fig. 2A). The acyl-CoA dehydrogenase (ACAD), which catalyzes the first rate-limiting step of FAO, has many different isomeric forms with different substrate specificities. In protein expression, several ACAD isomeric proteins are downregulated in CKD3/4 group versus control group (all *P* < 0.05, Fig. 2B). Protein expression of the mitochondrial trifunctional protein (MTP) beta subunit (HADHB) protein is reduced in CKD3/4 versus control, but the difference does not reach a statistical significance (*P* = 0.07, Fig. 2B).

Gene expression of several ACADs is downregulated in CKD groups compared to control group in the cortex (Fig. 2C) and medulla (Fig. 2D). There is also a downregulation in the gene coding for the 3-hydroxyacyl-CoA dehydrogenase (HADH) for medium-chain fatty acid (MCFA) and short-chain FA (SCFA) β-oxidation, and the genes for MTP alpha subunit (HADHA) and beta subunit (HADHB) for LCFA β-oxidation (Fig. 2C, D). Notably, in the medulla, expression changes of several genes take place in the early stages of CKD (CKD1/2), while in the cortex these changes occur in the late stages of CKD (CKD3/4).

Uptake of FA is mediated by fatty acid translocase (CD36) and fatty acid biding proteins (FABPs). FAs in the cytosol are transported into mitochondrial matrix by acyl-CoA synthetases (ACSs) and carnitine palmitoyltransferases (CPT1 and CPT2) for β-oxidation (Fig. 2A). In gene expression, a downregulation of several genes associated with FAT is observed in CKD3/4 in the cortex (all *P* < 0.05, Fig. 2E), and in both CKD1/2 and CKD3/4 in the medulla (all *P* < 0.05, Fig. 2F) compared with control. FABP1 and FABP3 proteins are also downregulated in CKD3/4 group in the cortex (Fig. 2G) and medulla (Fig. 2H) versus control (all *P* < 0.05). In addition, CPT1 and ACSM3 enzyme proteins are downregulated, while ACSL3 and ACSL4 are upregulated in CKD3/4 in the cortex (all *P* < 0.05, Fig. 2G).

In the circulation, many species of free FAs (FFAs) and FAO intermediates, including 3-hydroxy FAs (3-OH FAs) and fatty acyl carnitines (Fig. 2I) accumulate in cats with CKD when compared with control cats, signifying impaired FAO^24^, with the exception of 3-hydroxystearate (3OH C18) and capric acid (C10:0, red arrow). The concentration of capric acid, an MCFA, is reduced in both CKD groups compared to the control group.

While LCFAs and MCFAs are oxidized in the mitochondria, very long-chain FAs (VLCFAs) with 22 to 26 carbon units are almost exclusively oxidized in the peroxisome^25^. Peroxisomal VLCFA β-oxidation depends on the rate-limiting enzyme acyl-CoA oxidase 1 (ACOX1) and acetyl-CoA acyltransferase 1 (ACAA1) (Supplementary Fig. 3A). In the cortex and medulla, gene expression of *ACOX1* and *ACAA1* is downregulated in CKD versus control groups (Supplementary Fig. 3B, C). Expression of *ACOX3 gene*, which encodes an enzyme for branched-chain FA (BCFA) α-oxidation, is also downregulated in CKD (Supplementary Fig. 3B, C).

#### Evidence of disrupted renal glucose homeostasis in CKD

**Fig. 3:**
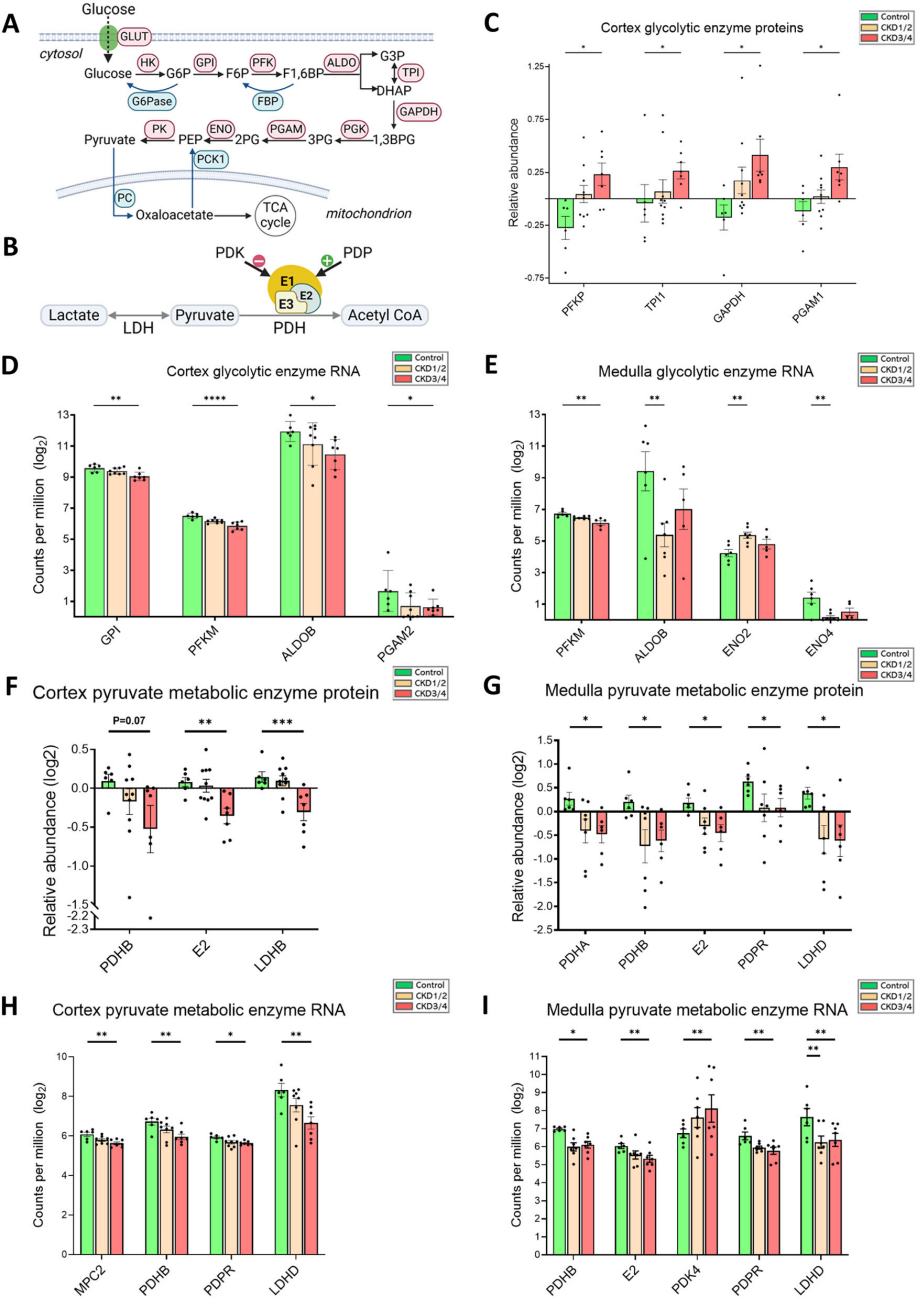
Evidence of disrupted renal glucose homeostasis in CKD. Schematics of (**A**) glucose metabolism and gluconeogenesis, and (**B**) pyruvate metabolism. **C** Protein expression of glycolytic enzymes in the cortex. **D**, **E** Expression of glycolytic genes in the cortex and medulla, respectively. **F**, **G** Protein expression of pyruvate metabolism enzyme proteins in the cortex and medulla, respectively. **H**, **I** Expression of genes coding for pyruvate metabolism enzymes in the cortex and medulla, respectively. Bar plots show means and SEM from the data. Negative values in protein expression are the results of log2 transformation of data <1. Sample size for RNA-seq experiments in the cortex (**D**, **H**): control (*n* = 6), CKD1/2 (*n* = 8), CKD3/4 (*n* = 7); in the medulla (**E**, **I**): control (*n *= 6), CKD1/2 (*n* = 7), CKD3/4 (*n* = 5); Sample size for proteomics experiments in the cortex (**C**, **F**): control (*n* = 6), CKD1/2 (*n* = 10), and CKD3/4 (*n* = 7); in the medulla (**G**): control (*n* = 6), CKD1/2 (*n* = 7), and CKD3/4 (*n* = 6). Error bars represent the SEM. *P < 0.05, **P < 0.01, ***P < 0.001, ****P < 0.0001. Panels (**A**, **B**) were created in BioRender.com. G6P glucose 6-phosphate, F6P fructose 6-phosphate, F1,6BP fructose 1,6-bisphosphate, G3P glyceraldehyde-3-phosphate, 1,3BPG 1,3-bisphosphoglycerate, 3PG 3-phosphoglycerate, 2PG 2-phosphoglycerate, PEP phosphoenolpyruvate, HK hexokinase, GPI glucose 6-phosphate isomerase, PFK phosphofructokinase, ALDO aldolase, TPI triosephosphate isomerase, GAPDH glyceraldehyde 3-phosphate dehydrogenase, PGK phosphoglycerate kinase, PGAM phosphoglycerate mutase, ENO enolase, PK pyruvate kinase, G6Pase glucose 6-phosphatase, FBP fructose 1,6-bisphosphatase, PCK1 phosphoenolpyruvate carboxykinase 1, PC pyruvate carboxylase, LDH lactate dehydrogenase, PDH pyruvate dehydrogenase, PDK pyruvate dehydrogenase kinase, PDP pyruvate dehydrogenase phosphatase, E1,E2,E3 pyruvate dehydrogenase complex subunits E1, E2, E3.

Glucose oxidation yields less ATP per molecule than FAO but requires less oxygen. Glucose metabolism generates pyruvate, which can be converted to acetyl-CoA to feed the TCA cycle and OXPHOS under aerobic conditions (Fig. 3A), or lactate under anaerobic conditions (Fig. 3B). In protein expression, several enzyme proteins, including a member of the phosphofructokinase (PFK) family (i.e. PFKP), triosephosphate isomerase 1 (TPI1), glyceraldehyde 3-phosphate dehydrogenase (GAPDH), and phosphoglycerate mutase 1 (PGAM1) are upregulated in CKD3/4 versus control in the cortex (Fig. 3C). In contrast, gene expression of aldolase B (ALDOB) and PFKM, another member of the PFK family, is downregulated in CKD3/4 in the cortex and in CKD1/2 in the medulla (all *P* < 0.05, Fig. 3D, E). While enolase 4 (ENO4) gene is downregulated in CKD1/2 in the medulla, ENO2 is upregulated (both *P* < 0.01, Fig. 3E).

Pyruvate dehydrogenase (PDH) complex (PDC) is composed of three catalytic components, namely, E1 (PDH), E2 (dihydrolipoamide transacetylase), and E3 (dihydrolipoamide dehydrogenase), and two regulatory components, PDH kinase (PDK) and PDH phosphatase (PDP) (Fig. 3B). The E1 enzyme is a heterotetramer of two alpha (PDHA) and two beta (PDHB) subunits. Protein expression of E2 is downregulated in the cortex and medulla (both *P* < 0.05, Fig. 3F, G, respectively), and that of PDHA and PDHB is downregulated in the medulla in CKD3/4 versus control (both *P* < 0.05, Fig. 3G). The level of PDHB protein is reduced in the cortex, but the change doesn’t reach a statistical significance (*P* = 0.07, Fig. 3F). In addition, protein expression of the LDH subunit B (LDHB) in the cortex (Fig. 3F) and lactate dehydrogenase D (LDHD) in the medulla (Fig. 3G) is downregulated in CKD3/4 versus control (*P* < 0.001 and *P* < 0.05, respectively). In gene expression, *PDHB* is downregulated in CKD3/4 in the cortex (*P* < 0.01, Fig. 3H) and medulla (*P* < 0.05, Fig. 3I), and *E2* is downregulated in the medulla (*P* < 0.01, Fig. 3I). The PDP activates PDC by dephosphorylating E1, while PDK phosphorylates E1, which in turn inactivates PDC (Fig. 3B). In CKD, *PDPR*, a PDP regulatory subunit, is downregulated in the cortex and medulla (*P* < 0.05 and *P* < 0.01, Fig. 3H, I, respectively), while *PDK4*, which is highly expressed in the kidney^26^, is upregulated in the medulla (*P* < 0.01, Fig. 3I). Finally, expression of *MPC2*, a pyruvate carrier and transporter, is downregulated in CKD3/4 in the cortex (*P* < 0.01, Fig. 3H).

The kidney is one of the two organs with sufficient activities of gluconeogenic enzymes, particularly glucose 6-phosphatase (G6Pase), for glucose synthesis and release^27^ (Fig. 3A, blue arrows). Gene expression of G6Pase catalytic subunit 1 (*G6PC1*), and fructose-1,6-bisphosphatases (*FBP1*, *FBP2*), is decreased in CKD3/4 in the cortex (all *P* < 0.01, Supplementary Fig. 4A), and in CKD1/2 in the medulla (*P* < 0.05, *P* < 0.001, *P* < 0.01, respectively, Supplementary Fig. 4B) versus control. Gene expression of mitochondrial pyruvate carboxylase (*PC*) is also downregulated in CKD1/2 versus control in the medulla (*P* < 0.01, Supplementary Fig. 4 B).

#### Evidence of altered ketone metabolism

**Fig. 4:**
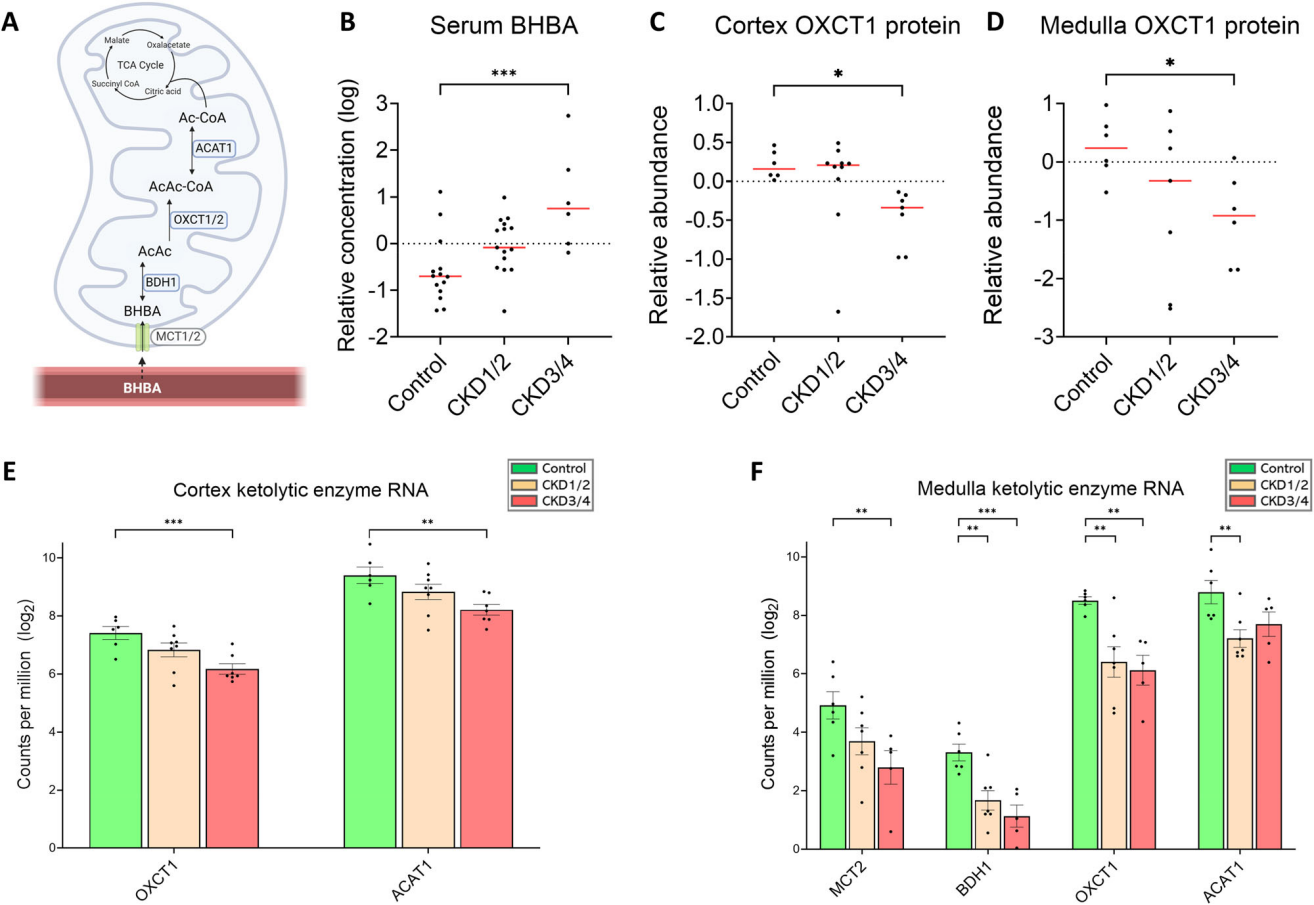
Evidence of impaired renal ketone oxidation. **A** Schematic of ketone metabolism. Update of ketones from the bloodstream (maroon) into extrahepatic mitochondria, where the ketones are oxidized to produce ATP. **B** Serum BHBA concentration. **C**, **D** OXCT1 protein abundance in the cortex and medulla, respectively. Negative values are the results of log2 transformation. **E**, **F** Expression of genes coding for ketolytic enzymes in the cortex and medulla, respectively. Sample size for metabolomics (**B**): control (*n* = 14), CKD1/2 (*n* = 15), and CKD3/4 (*n* = 6). Sample size for proteomic experiments in the cortex (**C**): control (*n* = 6), CKD1/2 (*n* = 10), and CKD3/4 (*n* = 7); in the medulla (**D**): control (*n* = 6), CKD1/2 (*n* = 7), and CKD3/4 (*n* = 6). Sample size for RNA-seq experiments in the cortex (**E**): control (*n* = 6), CKD1/2 (*n* = 8), CKD3/4 (*n* = 7); in the medulla (**F**): control (*n* = 6), CKD1/2 (*n* = 7), CKD3/4 (*n* = 5). Red bars in (**B**−**D**) represent the medians. Error bars in (**E**, **F**) represent the SEM. *P < 0.05, **P < 0.01, ***P < 0.001. Panel A was created in BioRender.com. OXCT1/2 3-oxoacid CoA-transferase gene family members 1 and 2, ACAT1 acetyl-CoA acetyltransferase 1, BHBA β-hydroxybutyric acid, MCT1/2 monocarboxylate transporter family members 1 and 2, BDH1 β-hydroxybutyrate dehydrogenase 1.

Monocarboxylate transporters (MCT1 and MCT2) play a crucial role in facilitating the uptake of ketones from the bloodstream into extrahepatic cells, where ketones undergo oxidation to produce ATP (Fig. 4A). The concentration of β-hydroxybutyric acid (BHBA), the main ketone body, is elevated in the blood in CKD3/4 versus control (*P* < 0.001, Fig. 4B). In protein expression, 3-oxoacid CoA-transferase 1 (OXCT1), catalyzing the first and rate-limiting step of ketolysis, is downregulated in CKD3/4 versus control in both cortex and medulla (both *P* < 0.05, Fig. 4C, D). Expression of OXCT1 gene is downregulated in CKD3/4 in the cortex (*P* < 0.001, Fig. 4E), and in both CKD groups in the medulla (both *P* < 0.01, Fig. 4F) compared to control. Additionally, expression of mitochondrial acetyl-CoA acetyltransferase 1 (ACAT1) gene is downregulated in CKD3/4 in the cortex and in CKD1/2 in the medulla (both *P* < 0.01, Fig. 4E, F). Further, there is a downregulation of ketone transporter MCT2 gene expression in CKD3/4, and 3-hydroxybutyrate dehydrogenase 1 (BDH1) in both CKD1/2 and CKD3/4 in the medulla when compared with control (all *P* < 0.01, Fig. 4F).

#### Evidence of impaired glutamine metabolism

Glutamine, another important energy substrate for the kidney, can be hydrolyzed to glutamate and ammonia by glutaminase (GLS) (Supplementary Fig. 5A). Glutamate enters the TCA cycle as α-ketoglutarate (α-KG)^12,28^, while ammonia, the main proton buffer in the urine, plays an important role in acid-base homeostasis^29^. Glutamine oxaloacetic transaminase 2 (GOT2) and glutamate-pyruvate transaminase 2 (GPT2) are mitochondrial transaminases that convert glutamate to α-KG. There is a downregulation of genes coding for GLS, GOT2, and GPT2 enzymes in CKD3/4 versus control in the cortex (all *P* < 0.05, Supplementary Fig. 5B), and in both CKD groups in the medulla (all *P* < 0.01, Supplementary Fig. 5C). In protein expression, there is a reduction of GOT2 and GPT2 enzyme proteins in CKD3/4 versus control in the cortex (both *P* < 0.05, Supplementary Fig. 5D, E), and GPT2 protein in both CKD groups versus control in the medulla (*P* < 0.05, Supplementary Fig. 5F).

#### Evidence of repressed tricarboxylic acid (TCA) cycle

**Fig. 5:**
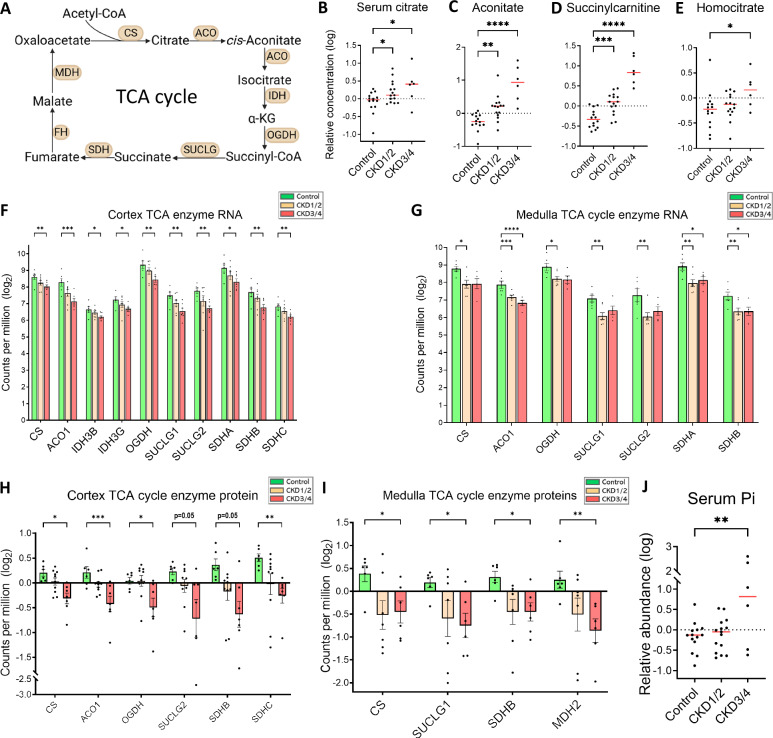
Repressed TCA cycle and OXPHOS. **A** Schematic of the TCA cycle. **B**−**E** Serum relative concentration of TCA cycle intermediates (citrate and aconitate) and metabolites (succinylcarnitine and homocitrate), respectively. **F**, **G** Expression of TCA cycle genes in the cortex and medulla, respectively. **H**, **I** Expression of TCA cycle enzyme proteins in the cortex and medulla, respectively. Negative values are the results of log2 transformation. **J** Serum concentration of inorganic phosphate (Pi). Sample size for metabolomics (**B**–**E**, **J**): control (*n* = 14), CKD1/2 (*n* = 15), and CKD3/4 (*n* = 6). Sample size for RNA-seq experiments in the cortex (**F**): control (*n* = 6), CKD1/2 (*n* = 8), CKD3/4 (*n* = 7); in the medulla (**G**): control (*n *= 6), CKD1/2 (*n* = 7), CKD3/4 (*n* = 5); Sample size for proteomics experiments in the cortex (**H**): control (*n* = 6), CKD1/2 (*n* = 10), and CKD3/4 (*n* = 7); in the medulla (**I**): control (*n* = 6), CKD1/2 (*n* = 7), and CKD3/4 (*n* = 6). Red bars represent the median. Error bars represent the SEM. *P < 0.05, **P < 0.01, ***P < 0.001, ****P < 0.0001. Panel A was created in BioRender.com. TCA tricarboxylic acid, OXPHOS oxidative phosphorylation, CS citrate synthase, ACO aconitase, IDH isocitrate dehydrogenase, OGDH 2-oxoglutarate dehydrogenase, SUCLG succinate-CoA ligase GDP-forming, SDH succinate dehydrogenase, FH fumarate hydratase, MDH malate dehydrogenase.

Under the physiological conditions, renal ATP production takes place in the mitochondria via OXPHOS, which utilizes reducing agents generated from the TCA cycle^15^ (Fig. 5A). In the bloodstream, TCA cycle intermediates such as citrate and aconitate (Fig. 5B, C), as well as TCA cycle metabolite succinylcarnitine (Fig. 5D), accumulate in CKD1/2 and CKD3/4 groups compared to control group (all *P* < 0.05). The concentration of circulating homocitrate^30^, another TCA cycle metabolite that is generated from acetyl-CoA and α-KG by homocitrate synthase, is also increased in CKD3/4 versus control (*P* < 0.05, Fig. 5E). Renal gene and protein expression of citrate synthase (CS), the first and rate-limiting enzyme of the TCA cycle, is downregulated in the cortex (Fig. 5F, H) and in the medulla (Fig. 5G, I) in CKD versus control (all *P* < 0.05). In the cortex, there is a downregulation in gene expression of aconitase 1 (*ACO1*), NAD^+^-dependent isocitrate dehydrogenase 3 subunits (*IDH3B* and *IDH3G*), oxoglutarate dehydrogenase (*OGDH*), succinate-CoA ligase (*SUCLG*) subunits (*SUCLG1* and *SUCLG2*), succinate dehydrogenase complex (SDH) components (*SDHA*, *SDHB*, and *SDHC*) in CKD3/4 versus control (all *P* < 0.05, Fig. 5F). Similarly in the medulla, there is a downregulation in gene expression of *ACO1*, *OGDH*, *SUCLG1*, *SUCLG2*, *SDHA*, and *SDHB* in CKD1/2 versus control, and *ACO1*, *SDHA* and *SDHB* in CKD3/4 versus control (all *P* < 0.05, Fig. 5G). At the protein level, expression of ACO1, OGDH, SUCLG2, SDHB and SDHC enzyme proteins in the cortex (Fig. 5H), and expression of SUCLG1 and SDHB in the medulla (Fig. 5I) is reduced in CKD3/4 versus control (all *P* ≤ 0.05). Expression of mitochondrial malate dehydrogenase (MDH2) protein is also reduced in the medulla (*P* < 0.01, Fig. 5I).

The concentration of inorganic phosphate (Pi), substrate of ATP synthase, is increased in the bloodstream in CKD3/4 versus control (Fig. 5J).

### Disturbed redox homeostasis and impaired mitophagy

**Fig. 6:**
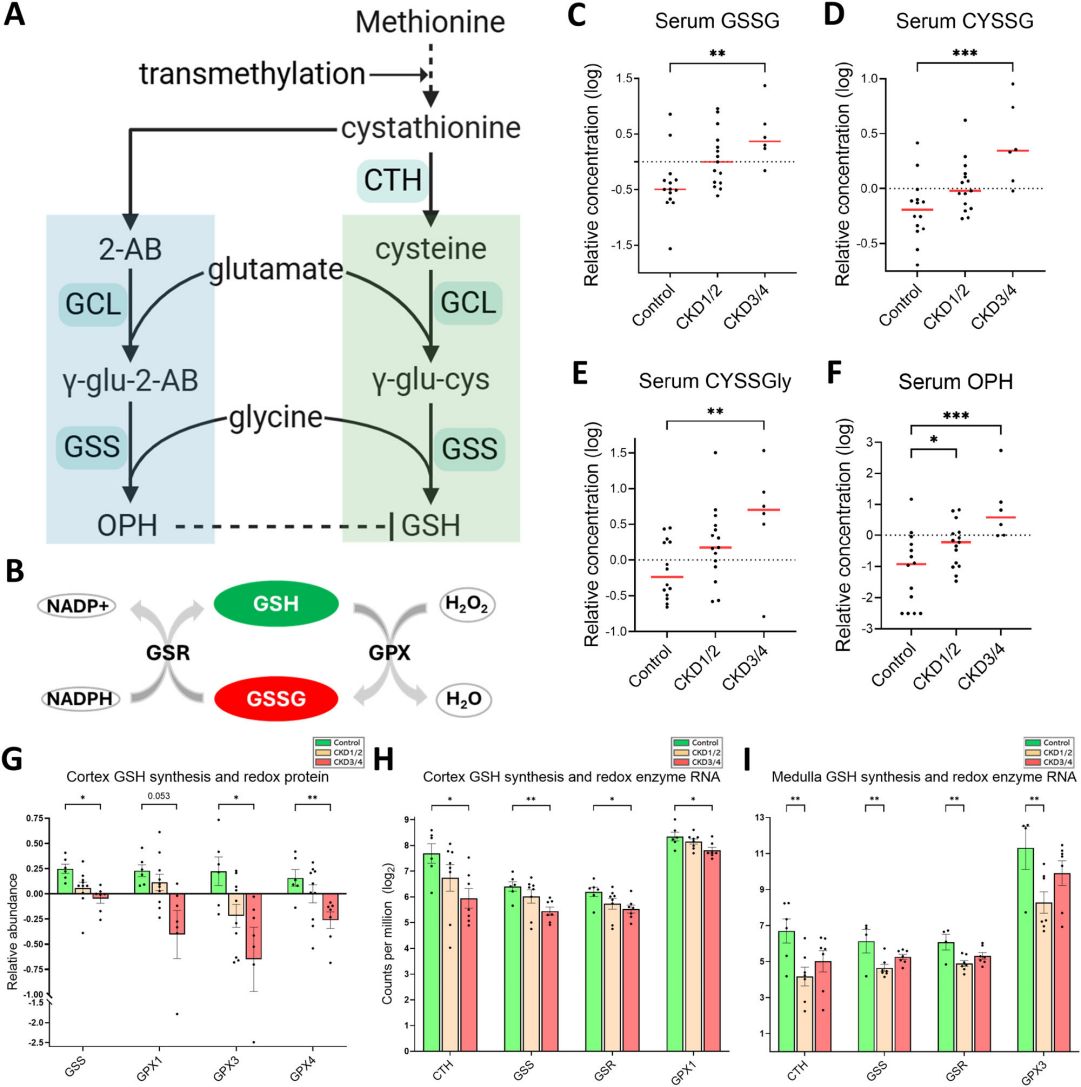
Evidence of disturbed redox homeostasis. Schematic of (**A**) GSH (green) and OPH (blue) synthesis pathways, and (**B**) redox cycle. **C**−**F** Serum concentration of GSSG, CYSSG, CYSSGly, and OPH, respectively. **G** Protein expression of GSS and GPXs. Negative values are the results of log2 transformation. **H**, **I** Gene expression of *CTH*, *GSS*, *GSR*, and *GPX*s in the cortex and medulla, respectively. Sample size for metabolomics (**C**–**F**): control (*n* = 14), CKD1/2 (*n* = 15), and CKD3/4 (*n* = 6). Sample size for proteomic experiments in the cortex (**G**): control (*n* = 6), CKD1/2 (*n *= 10), and CKD3/4 (*n* = 7). Sample size for RNA-seq experiments in the cortex (**H**): control (*n* = 6), CKD1/2 (*n* = 8), CKD3/4 (*n* = 7); in the medulla (**I**): control (*n* = 6), CKD1/2 (*n* = 7), CKD3/4 (*n* = 5). Red bars represent the median. Error bars represent the SEM. *P < 0.05, **P < 0.01, ***P < 0.001. Panel A was created in BioRender.com. GSH reduced glutathione, GSSH oxidized glutathione, CYSSG cysteine-glutathione disulfide, CYSSGly cysteinylglycine disulfide, 2-AB α-aminobutyrate, OPH ophthalmic acid, CTH cystathionine γ-lyase, GSS glutathione synthetase, GCL glutamate-cysteine ligase, GSR glutathione-disulfide reductase, GPX glutathione peroxidase.

Glutathione (GSH) plays a crucial role in cellular redox homeostasis (Fig. 6A). It neutralizes reactive oxygen species (ROS) via cycling between reduced (GSH) and oxidized (GSSG) forms (Fig. 6B). Serum concentrations of oxidized glutathione (GSSH), cysteine-glutathione disulfide (CYSSG), and cysteine-glycine disulfide (CYSSGly) are increased in CKD3/4 compared to control (all *P* < 0.01, Fig. 6C–E). Ophthalmic acid (OPH), an endogenous GSH analog synthesized by the same enzymes involved in GSH biosynthesis (Fig. 6A, blue panel), is believed to act as a competitive inhibitor of GSH synthesis and function^31^. The concentration of OPH in the circulation is higher in both CKD groups versus control group (both *P* < 0.05, Fig. 6F).

At the protein level, glutathione synthetase (GSS) protein and several members of glutathione peroxidase (GPX) family (i.e. GPX1, GPX3 and GPX4) proteins are downregulated in CKD3/4 versus control in cortex (all *P* < 0.05, Fig. 6G). In gene expression, there is a downregulation in genes coding for GSS, GPX, cystathionine gamma-lyase (CTH), and glutathione-disulfide reductase (GSR) in CKD3/4 in the cortex (all *P* < 0.05, Fig. 6 H) and in CKD1/2 in the medulla (all *P* < 0.01, Fig. 6 I), compared to control.

The cell removes damaged and dysfunctional mitochondria to preserve mitochondrial integrity and function through the PINK1-Parkin-mediated mitophagy pathway^32^. Expression of gene encoding PINK1, a sensor for mitochondrial damage, is downregulated in CKD3/4 in the cortex and CKD1/2 in the medulla (both *P* < 0.01, Supplementary Fig. 6A, B). Similarly, there is a downregulation in the gene coding for parkin (PRKN), a ubiquitin protein ligase, in both CKD1/2 and CKD3/4 groups versus control group (all *P* < 0.05, Supplementary Fig. 6C, D).

### Increased renal fibrosis and inflammation in CKD

**Fig. 7:**
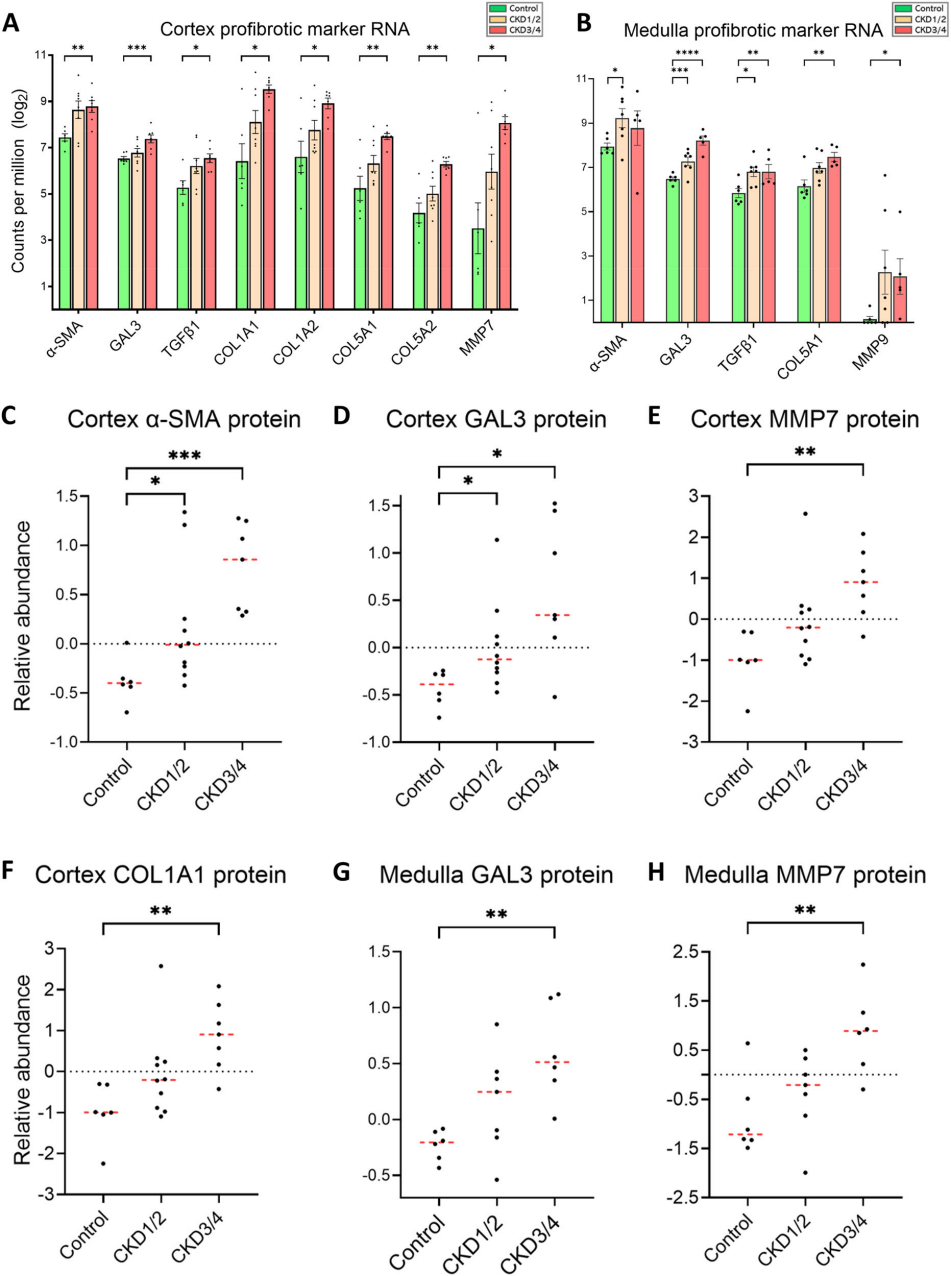
Profibrotic markers. **A**, **B** Gene expression of profibrotic markers in the cortex and medulla, respectively. **C**−**F** Cortical protein expression of α-SMA, GAL3, MMP7 and COL1A1, respectively. **G**, **H** Medullary protein expression of GAL3 and MMP7, respectively. Negative values are the results of log2 transformation. Sample size for RNA-seq experiments in the cortex (**A**): control (*n* = 6), CKD1/2 (*n* = 8), CKD3/4 (*n* = 7); in the medulla (**B**): control (*n* = 6), CKD1/2 (*n* = 7), CKD3/4 (*n* = 5). Sample size for proteomic experiments in the cortex (**C**−**F**): control (*n* = 6), CKD1/2 (*n* = 10), and CKD3/4 (*n* = 7); in the medulla (**G**, **H**): control (*n* = 6), CKD1/2 (*n* = 7), and CKD3/4 (*n* = 6). Red bars represent the median. Error bars represent the SEM. *P < 0.05, **P < 0.01, ***P < 0.001, ****P < 0.0001. α-SMA alpha-smooth muscle actin, GAL3 galectin 3, MMP7/9 matrix metallopeptidases 7 and 9, COL1A1/2 collagen type I alpha 1 and 2 chains, COL5A1/2 collagen type V alpha 1 and 2 chains, TGFβ1 transforming growth factor beta 1.

There is an upregulation of genes coding for alpha-smooth muscle actin (α-SMA), galectin 3 (GAL3), and matrix metallopeptidase 7 (MMP7) proteins in CKD3/4 versus control in the cortex, and those for α-SMA, GAL3, and MMP9 proteins in the medulla (Fig. 7A, B). Similar observation is made on genes such as collagen type I α and β chains (*COL1A1* and *COL1A2*) and collagen type V α and β chains (*COL5A1* and *COL5A2*) (Fig. 7A, B). In the protein expression level, there is an upregulation in α-SMA, GAL3, MMP7, and COL1A1 proteins in CKD versus control in the cortex (all *P* < 0.05, Fig. 7C–F), and GAL3 and MMP7 proteins in the medulla (both *P* < 0.01, Fig. 7G, H).

Gene expression of *NFκB* subunits (*NFκΒ1* and *NFκB2*) in the cortex (both *P* < 0.05, Supplementary Fig. 7A) and *NFκB2* in the medulla (*P* < 0.001, Supplementary Fig. 7B) is upregulated in CKD3/4 versus control. In addition, there is an upregulation of *NLRC5* and *NLRP3* in CKD3/4 versus control in the cortex (both *P* < 0.01, Supplementary Fig. 7A) and *NLRP3* in CKD3/4 in the medulla (*P* < 0.0001, Supplementary Fig. 7B). Interleukin 11 (*IL11*) and 16 (*IL16*) gene expression is also upregulated. Notably, protein expression of NFKB1 and IL16 is upregulated in both CKD1/2 and CKD3/4 in the cortex (all *P* < 0.05, Supplementary Fig. 7C, D), and IL16 protein level is increased in CKD3/4 in the medulla (*P* < 0.05, Supplementary Fig. 7E), when compared with the control. In addition, expression of profibrotic marker genes lysyl oxidase (*LOX*) and fibronectin 1 (*FN1*) is upregulated in both CKD1/2 and CKD3/4 in the cortex compared to control (all *P* < 0.05, Supplementary Fig. 7F, G). Endothelin 1 (*EDN1*) is upregulated in CKD3/4 in the cortex (*P* < 0.001, Supplementary Fig. 7H), and in both CKD1/2 and CKD3/4 in the medulla (both *P* < 0.05, Supplementary Fig. 7 I).

Finally, the correlation of expression among key profibrotic genes (Supplementary Fig. 8A) and between redox proteins and profibrotic protein markers (Supplementary Fig. 8B) in the cortex is examined. There is a strong and positive correlation of gene expression among profibrotic genes transforming growth factor beta 1 (*TGFβ1*), *COL1A1*, *COL1A2*, *COL5A1*, *COL5A2*, fibronectin 1 (*FN1*), and *MMP2*, *MMP7*, and *LOX* (all *P* < 0.0001, ρ > 0.75, Supplementary Fig. 8A, yellow box), which also show a moderate correlation with *GAL3* and *α-SMA* (all *P* < 0.01, *ρ* > 0.5). Protein expression of redox enzyme GSS is strongly and negatively correlated with that of MMP7, GAL3, and α-SMA (all *P* < 0.0001, *ρ* < -0.75, Supplementary Fig. 8B), while GPX3 and GPX4 show a moderate and negative correlation with MMP7, GAL3, and α-SMA.

### Evidence of impaired oxygen homeostasis

**Fig. 8:**
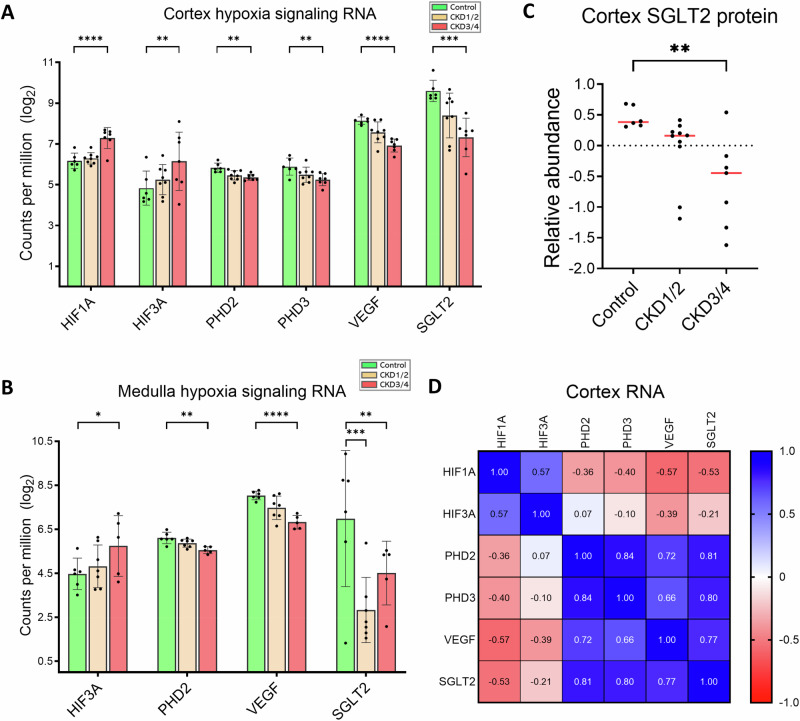
Activation of hypoxia signaling pathway in CKD. **A**, **B** Expression of genes encoding hypoxia signaling pathway enzymes in the cortex and medulla, respectively. **C** Cortical SGLT2 protein expression. Negative values are the results of log2 transformation. **D** Heatmap of Pearson’s correlation of RNA expression in the cortex. Blue and red indicate positive and negative correlations, respectively. The numbers in the heatmap indicate correlation coefficients. Sample size for RNA-seq experiments in the cortex (**A**): control (*n* = 6), CKD1/2 (*n* = 8), CKD3/4 (*n* = 7); in the medulla (**B**): control (*n* = 6), CKD1/2 (*n* = 7), CKD3/4 (*n* = 5). Sample size for proteomics experiments in the cortex (**C**): control (*n* = 6), CKD1/2 (*n* = 10), and CKD3/4 (*n* = 7). Red bars represent the medians. Error bars represent the SEM. *P < 0.05, **P < 0.01, ***P < 0.001, ****P < 0.0001. HIF1A hypoxia inducible factor 1 alpha, HIF3A hypoxia inducible factor 3 alpha, PHD2/3 prolyl hydroxylase domain- containing proteins 2 and 3, VEGF vascular endothelial growth factor, SGLT2 sodium-glucose cotransporter 2.

Hypoxia is an intrinsic adaptive response to low blood oxygen levels. Hypoxia-inducible factors (HIFs) are a group of transcription regulators that are targeted for degradation under normoxic conditions, while prolyl hydroxylase domain-containing proteins (PHDs) are oxygen sensors that stabilize and protect HIFs from degradation in low oxygen conditions^33^. In the cortex, there is an upregulation of HIF 1 alpha subunit (*HIF1A*) and HIF 3 alpha subunit (*HIF3A*) genes, but downregulation of *PHD2*, *PHD3*, and vascular epithelial growth factor (*VEGF*) genes in CKD3/4 versus control (all *P* < 0.01, Fig. 8A). In the medulla, expression of *HIF3A* is upregulated, while that of *PHD2* and *VEGF* is downregulated in CKD3/4 versus control (*P* < 0.05, *P* < 0.01, and *P* < 0.0001, respectively, Fig. 8B). Further, expression of sodium-glucose cotransporter 2 gene (*SGLT2*), responsible for the reabsorption of filtrated glucose in the PTCs, is downregulated in CKD3/4 in the cortex (*P* < 0.001, Fig. 8A) and in both CKD1/2 and CKD3/4 in the medulla (both *P* < 0.01, Fig. 8B). In the cortex, SGLT2 protein expression has a 1.8-fold decrease in CKD3/4 versus control (*P* < 0.01, Fig. 8C).

No expressional change in HIF2A or erythropoietin (EPO) is observed.

Finally, the relationships between *SGLT2* gene and several genes of the hypoxia signaling pathway in the cortex are explored. *SGLT2* expression is positively correlated with that of *PHD2*, *PHD3*, and *VEGF* (all *P* < 0.0001, *ρ*$$ > 0.75$$, Fig. 8D), but negatively with *HIF1A* (*P* = 0.01, *ρ*$$=-0.53$$, Fig. 8D). No correlation between *SGLT2* and *HIF2A* or *HIF3A* is observed (both *P* > 0.05).

## Discussion

Concomitant transcriptomic and proteomic analyses reveal spatiotemporal changes in renal tissue gene and protein expression. In the mammalian kidneys, the partial pressure is ~35–50 mmHg in the cortex but only 10−20 mmHg in the medulla^34^. Compared to the cortex, the medulla operates at the lower baseline oxygen levels, and is responsible for high metabolic activities such as active transport in the Loop of Henly, which are energy-intensive and require a large amount of oxygen, making renal medulla more susceptible for further oxygen reduction and vulnerable to ischemic injury. The observation that gene and protein expression changes occur earlier (i.e. CKD1/2) in the medulla than in the cortex may be, in part, due to the differences in the vascular anatomy and metabolic activities between the two regions. Accumulating evidence indicates that hypoxia plays a crucial role in the progression of CKD^13^. Hypoxia, a condition of insufficient oxygen supply to the kidneys, activates a myriad of expression of genes involved in fibrosis, oxidative stress, inflammatory response, angiogenesis, and bioenergetics^13,18^. The initial adaptive metabolic shift becomes maladaptive, leading to long-term kidney damage. Renal injury can also increase the needs of tissue repair, which stimulates aerobic glycolysis to provide intermediates for cellular proliferation^13,14^.

The kidney is a metabolic organ with a high mitochondrial density in the renal tubules^10^, and a major consumer of molecular oxygen^15^. The energy substrate preference varies along nephron and is largely driven by oxygen delivery. Under physiological conditions, the PTCs in the cortex metabolize FAs and amino acids via OXPHOS to support its high ATP demands for filtrate reabsorptions^15^. After injury, the kidney cells undergo metabolic reprogramming to meet the high energy demands that are crucial for cellular repair and recovery. The mechanisms by which the kidney shifts its energetic program in CKD are incompletely understood in humans and have not been explored in cats. The study provides multi-omic evidence that the kidney’s abilities to transport FFAs into cytoplasm and mitochondria and metabolize FAs is impaired in CKD. These alterations in FAT and FAO are evidenced as many FFAs and FAO intermediates accumulate in the circulation in CKD. In the medulla, there is a downregulation of genes involved in FAT (such as *FABPs*, *CD36*, and *ACSs*) and FAO (such as *ACADs, HADHA*, and *HADAB*) in the early stages of CKD. But in the cortex, downregulation of these genes occurs in the later stages of CKD (i.e. CKD3/4). Early disruption in FAO in the medulla may reflect the different sensitivity to oxygen tension between cortex and medulla whose unique microenvironment makes it more susceptible to metabolic disturbances. Alternatively, the spatial and temporal differences may suggest a sequential response where early changes in the medulla precede and signal subsequential metabolic adaptations in the cortex.

Very long-chain FAs with chain length of 22 carbons or longer undergo β-oxidation almost exclusively in the peroxisomes, resulting in the production of acetyl-CoA and medium-chain acyl-CoAs. Since the peroxisomes lack the enzymes necessary for the TCA cycle, MCFAs derived from peroxisomal VLCFA β-oxidation are transported into mitochondria^35^. The study provides evidence that VLCFA β-oxidation in peroxisomes is impaired in CKD, supporting the hypothesis that a disruption of peroxisomal VLCFA oxidation may lead to a reduced level of circulating MCFAs, such as capric acid.

Ketone bodies are preferred energy substrates for the distal segments of nephron performing active transport under physiological conditions^36^. But how ketone metabolism changes in CKD is not very well understood. In cats with CKD, BHBA, the main ketone body, accumulates in the circulation, and protein expression of OXCT1, the rate-limiting enzyme for ketone metabolism, is downregulated in both renal cortex and medulla. In gene expression, there is as well a downregulation of both *BDH1* and *OXCT1* in CKD1/2 in the medulla, but the change occurs in CKD3/4 in the cortex. Glutamine is the most abundant amino acid in the circulation of cats^37^ and people^38^. In the kidney, GLS catalyzes the hydrolysis of glutamine to ammonia and glutamate. The kidney is one of the key organs that regulate and maintain systemic acid-base balance by producing ammonia, the main proton buffer in the urine^29^. Venous pH was lower in cats with severe, but not mild or moderate, chronic renal failure (CRF), compared to healthy cats^39^.Urine pH was decreased in feline patients with increasing severity of CRF, and a reduction of urine ammonia to creatinine ratio was already observed in cats with stage 2 CKD vs. healthy cats^39,40^. Taken together, our observation along with previous ones suggests an important role of urine ammonia in regulating systemic acid-base balance in cats. Glutamate, on the other hand, can be further metabolized to produce α-KG by transaminases, GOT2 and GPT2, to feed the TCA cycle. Notably, a spatial and temporal regulation in the expression of genes (*GLS*, *GOT2*, *GPT2*) and proteins (GOT2, GPT2) is observed. Taken together, the data provides evidence of reduced renal energy production via glutamine and ketone oxidation, and a link between impaired glutamine metabolism and acid-base imbalance in feline CKD.

Glucose contributes to little ATP production in the PTCs of the cortex but is a preferred substrate for glomeruli and distal parts of nephron in the medulla^15^. In a recent study to evaluate glucose metabolism using mouse subtotal nephrectomy model of CKD, gene expression of many glycolytic enzymes is upregulated in the PT of the nephrectomized kidney^16^. In contrast, the transcriptomic analysis shows a downregulation of genes associated with glucose metabolism in both cortex and medulla in CKD. The phosphorylation/dephosphorylation cycle of PDH by PDK/PDP leads to its inhibition/activation, respectively, serving as the gatekeeper for glycolysis and pyruvate metabolism. The study provides evidence that gene and protein expression of PDC catalytic components, PDHA, PDHB, and E2, is downregulated. Expression of *PDK4* gene is upregulated, while that of PDP regulatory subunit (*PDPR*) is downregulated in CKD. Intriguingly, an upregulation in protein expression of several glycolytic enzymes (such as PFKP, TPI1, PGAM1, and GAPDH) in the cortex, but not in the medulla, is observed, suggesting a differential post-translational regulation to increase glucose metabolism in the cortex. Because the TCA cycle is repressed in CKD, it is hypothesized that the kidney upregulates cortical enzymes in the glycolytic pathway to provide glycolytic intermediates for shunting pentose phosphate pathway (PPP) towards the synthesis of nucleotides and other macromolecules for rapid cell proliferation and repair, rather than for energy production. Targeting specific enzymes within the PPP may offer an opportunity for novel pharmaceutic or nutritional intervention for feline CKD. Importantly, the interconversion between pyruvate and lactate is altered, further exacerbating energy deficit.

The kidney is one of the only two organs possessing sufficient gluconeogenic enzyme activities besides the liver, thus playing a significant role in glucose homeostasis in humans and animals^27^. It was postulated that renal gluconeogenesis is confined to the PT, which is the only nephron segment with required enzyme activities^41^. However, the transcriptomic data show that several genes in the gluconeogenic pathway, including *G6Pases*, are expressed at similar levels in both cortex and medulla, and their expression is downregulated in CKD. Nevertheless, there is a possibility of differential translational regulations between the two regions. Two hypoxia inducible isoforms, HIF1A and HIF2A, both participate in the response to hypoxia^42,43^. While activation of HIF1A increases inflammation, fibrosis and tubular tissue damage, HIF2A exerts anti-inflammatory, and anti-fibrotic effects. The HIF1A regulates the transcription of genes coding for metabolic enzymes, transporters, and mitochondrial proteins to reduce oxygen consumption, and HIF2A is responsible for EPO synthesis. It is postulated that the shift in the balance of HIF1A/HIF2A contributes to the progression of diabetic kidney disease (DKD) in humans^42,43^. In cats with CKD, no expressional change in HIF2A or EPO is observed. Possibly, the discrepancy lies with the different risk factors between the two species, where diabetes is not a significant risk factor for feline CKD. SGLT2, which is localized in the PT, is responsible for >97% reabsorption of filtered glucose from the urine^44^, a process that consumes a significant amount of oxygen. SGLT2 inhibitors (SGLT2i) provides nephroprotective benefits in to human patients with DKD, presumably by suppressing HIF1A and stimulating HIF2A, which in turn augments erythropoiesis^44–47^. In this study, expression of SGLT2 protein is decreased by nearly 2-fold in CKD in the cortex, suggesting reduced tubular glucose reabsorptions and oxygen consumptions. Recently, the US Food and Drug Administration (FDA) approved bexagliflozin, a SGLT2i, to treat diabetic cats^48^. Despite the differences, many of the mechanisms by which SGLT2i exert their effects in human kidney diseases might also apply to cats beyond their use to treat diabetes, which will be determined by future clinical trials^49^. In general, hypoxia stimulates the production of VEGF protein, leading to angiogenesis and protection against ischemic injury^50^. Surprisingly, gene expression of *HIF1A* is upregulated, yet *VEGF* gene is downregulated. However *VEGF* gene expression is strongly and positively associated with *SGLT2* expression. It is possible that a decrease in *VEGF* gene expression is a result of reduced tubular oxygen consumption due to reduced glucose reabsorption. The intricate and complex interplay between glucose homeostasis, hypoxia and neovascularization warrants further investigation.

Tissue fibrogenesis reflects the sequelae of injury or prolonged inflammatory events, and the resultant disruption and graduate destruction of tissue architecture and function^9^. Myofibroblasts, which are differentiated from interstitial fibroblasts, express α-SMA and produce extracellular matrix (ECM) proteins such as collagen and fibronectin^51^. The excessive deposition of ECM proteins in the interstitial spaces contributes to renal fibrosis. The initial phases of fibrogenesis includes activation of macrophages and cytokines, including TGFβ, and dissolution of matrix, followed by production and deposition of matrix proteins such as fibronectin and collagens. TGFβ1 is thought to be the master regulator of renal fibrosis, particularly in CKD^52^. TGFβ1 signaling stimulates the transcription of a cascade of genes related to ECM production, including collagens and fibronectin, and activation of myofibroblasts, which express α-SMA^51,52^. Protein expression of α-SMA, COL1A1, and MMP7 proteins is upregulated in CKD vs. control in the cortex, MMP7 metallopeptidase in the medulla. In gene expression, *TGFβ1* is strongly and positively correlated with *FN1*, *LOX*, *MMP2*, *MMP7*, and *collagens type I* and *V*. Remarkably, many of these profibrotic genes have a correlation coefficient equal to or greater than 0.90 with one another. Expression and secretion of GAL3 by macrophages is thought to play a role in the activation of renal fibroblasts to its profibrotic prototype^53^, and elevated plasma GAL3 is associated with major adverse kidney events and death^54^. To the contrary, a study using a rodent model of induced CKD showed GAL3 protects renal tubular injury by limiting apoptosis and attenuates fibrosis^55^. In cats with naturally occurring CKD, expression of GAL3 protein is upregulated in both cortex and medulla, while its gene expression is positively correlated with that of profibrotic genes, suggesting a role of GAL3 in renal fibrogenesis. NFκB, a transcription factor, is a pivotal mediator of inflammatory responses, while inflammasomes, such as members of NOD-like receptor family, including NLRC5 and NLRP, play a key role in sensing inflammatory signals and triggering the innate immune response^56^. Upregulations of NFκB and inflammatory signaling provide evidence of elevated inflammatory responses in the kidneys of cats with CKD.

Mitochondrial OXPHOS contributes to ~90% of cellular reactive oxygen species (ROS) production, which under physiological conditions, is finely regulated with an array of antioxidants, many of which depend on GSH^57^. The redox cycle between reduced GSH and oxidized GSSG is central to this process. Indeed, GSSG, CYSSG, and CYSSGly are hallmarks of oxidative stress, and their concentrations in the blood are increased in CKD. In the kidney, expression of GSS, GSR and GPXs genes and proteins, is downregulated in CKD. A recent proteome study connected GPX3, ROS generation and kidney fibrosis in CKD^20,21^. GPX3 protects renal tubular cells from oxidative damage and the resulting inflammation. A downregulation of GPX3 may induce kidney fibrosis via developing fibrotic extracellular matrix^20^. A significant negative correlation between redox enzymes, GSS and GPXs, and profibrotic markers, MMP7, GAL3, and α-SMA, indicates a connection between redox homeostasis and fibrosis in cats with CKD.

The study includes paired transcriptomic and proteomic data from kidney tissues of control cats and cats with early and late stages of CKD, as well as serum metabolomic data from a separate cohort of cats. The findings are consistent across groups despite of small sample size. There are some important limitations in the current study. The cats in cohort 1 were significantly different in age and body weight between control and CKD groups. Although indirect blood pressure was measured in cats in cohort 1, cats in cohort 2 had their blood pressure measured only if their health permitted due to ethical considerations. It is thus possible systemic hypertension, common in feline CKD, might confound the transcriptomic and proteomic findings. Serum metabolomic profiling reflects systemic changes of metabolites, which may be influenced by other factors such as diet, medications, or comorbidities. Typically, cats with CKD are on specialized diets that are formulated to support kidney function. Additionally, many cats with CKD experience decreased appetite and weight loss, so it is crucial to encourage food consumption by offering different food types, including dry, wet or both. Medications, including those for CKD management, can also influence global metabolome changes. Moreover, cats with subclinical conditions, such as subclinical cancer or heart disease, might be included. Another limitation is that the majority of cats in cohort 1 are not part of cohort 2, preventing direct correlation analysis between systemic metabolome and tissue transcriptome and proteome. This is likely to be addressed in future studies with a large cohort of cats. Renal tissue metabolomics should be performed in the future to understand metabolome changes in the kidney tissue and to corroborate tissue transcriptome and proteome changes. Due to a high cellular heterogeneity in the kidneys, single-cell RNA sequencing technology will offer a significant advantage over the traditional RNA sequencing by providing a high-resolution transcriptomic atlas with specific cell types and their associations with pathophysiology, including metabolic reprogramming, fibrogenesis, and inflammation, in feline CKD. Finally, CKD in cats is heterogeneous in the rate of progression, which is not accounted for in this cross-sectional study. The study, despite the limitations, utilizes multi-omics analysis to gain initial insights into the complexity of renal metabolism, providing a detailed depiction of metabolic landscape of feline CKD.

## Methods

### Animals and study design

The study protocol was reviewed and approved by the Institutional Animal Care and Use Committee of the Nestlé Purina PetCare Company (approval numbers NT7427 and NT7749). We have complied with all relevant ethical regulations for animal use. Two cohorts of cats were enrolled for the study. The cohort 1 consisted of 35 domestic short-hair cats from the same research colony (Supplementary Table 1), including 14 healthy control cats, 15 CKD cats with International Renal Interest Society (IRIS) stage 1 or stage 2 (CKD1/2), and 6 CKD cats with IRIS stage 3 or stage 4 (CKD3/4). There were 22 females and 13 males with the mean ages of 10.9, 14.9 and 11.7 years for the control, CKD1/2, and CKD3/4 groups, respectively. The cohort 2 included 19 domestic short-hair and 4 domestic long-hair cats that were euthanized for humane reasons unrelated to the study (Supplementary Table 2). There were 14 females and 9 males with the mean ages of 13.7, 14.3, and 12.8 years for the control, CKD1/2, and CKD3/4 groups (*P* = 0.85), respectively. Renal cortical and medullary tissues were collected and stored in the RNA*later* solution (Thermo Fisher Scientific Inc.) until use. Cats with major systemic diseases such as cancer, hyperthyroidism, heart failure, or diabetes mellitus were excluded from the study.

### Metabolomics, RNA-seq, and proteomics

Serum samples were submitted for untargeted metabolomic analysis in a commercial laboratory (Metabolon, Inc.). Sample preparation and extraction, liquid chromatography, MS, compound detection and identification, and statistical analysis were described previously^58^ and available in Supplementary Method 1. Briefly, the raw data (Supplementary Data 2) are generated based on the area-under-the-curve formula using ion counts that provide relative quantification. Metabolites with >80% missing values are removed. For each metabolite, missing values are replaced 20% of its observed minimum. The data are then transformed using the natural log, and autoscaled to achieve a zero mean and unit variance for all metabolites. A multiple linear regression model adjusted for age and sex was applied to the normalized and log transformed data. *P*-values were adjusted to control the false discovery rate (FDR).

Total RNA was extracted from both renal tissues and a cDNA library was constructed. RNA samples with poor RNA integrity numbers were removed. The libraries were quantified to be pooled equimolarly and sequenced on an Illumina NextSeq 2000 with V3 chemistry pair-end 150 cycles to achieve sequencing depth ~100 million reads per sample. Samples with <100 million reads or an average read length shorter than 200 nucleotides were removed, resulting in 21 cortical and 18 medullary samples. The fastq files were created and demultiplexed using casava v1.8.2 from the raw sequencing data, aligned against the domestic cat reference genomes Felis_catus_9.0 using RNAstar v2.5.3. The htseq_count v2.16.2 program was used to quantify the counts and genes were filtered when there were <2 counts per million in ≥5 samples. The normalization method was the trimmed mean of M-values method performed by the function calcNormFactors in edgeR. The edgeR package function glmQLFTest was also used to fit a quasi-likelihood negative binomial generalized log-linear model. More details can be found in Supplementary Method 2. The raw read counts of gene expression data can be found in Supplementary Data 3–4.

The MS-based proteomic analysis is detailed in Supplementary Method 3. Briefly, renal tissues were lysed in RIPA buffer (Sigma-Aldrich) conditions using bead beating. After extraction, protein samples were reduced, alkylated, precipitated, and digested with trypsin/Lys-C mix (Promega). Resulting peptides were labeled with tandem mass tag for quantification purposes. Samples were analyzed with liquid chromatography (LC)-tandem MS (MS/MS) using an Orbitrap Fusion Lumos (Thermo Scientific). Protein identifications were obtained using Mascot 2.6.1 (Matrix Science) against the UniProt Felis catus reference proteome database. Scaffold (Proteome Software) was used for validation and visualization of the data. Triplicate quantitative values, expressed as log2 fold changes with regards to a reference pool of each tissue type, were averaged for each protein and sample. Following sample and data quality control steps, 23 cortical and 19 medullary samples remained. A linear model was fitted to each protein adjusting for sex and compared each CKD groups with control. The *P*-values associated from the linear models were adjusted for multiple testing error using the Benjamini-Hochberg method. The protein expression data can be found in Supplementary Data 5–6.

In many clinical settings, FDR < 0.05 has been suggested as being too low to embrace all potential meaningful results^59,60^. In some studies, less restricted statistical approaches, including *P* < 0.05, were proposed and applied^61,62^. Combining paired RNA and protein-level data enables corroboration between the two independent measurements, substantiating the results obtained from individual datasets^63^. In the context of multi-omics analysis, a threshold of FDR < 0.10 for differentially expressed metabolites and genes, and FDR < 0.30 for differentially expressed proteins is applied.

### Statistics and reproducibility

For serum metabolomics experiment, there were 35 cats divided into three groups: healthy control (*n* = 14), CKD1/2 (*n* = 15) and CKD3/4 (*n* = 6). For RNA-seq experiment, cortical tissues were collected from control (*n* = 6), CKD1/2 (*n* = 8), and CKD3/4 (*n* = 7) cats, and medullary tissues from control (*n* = 6), CKD1/2 (*n* = 7), CKD3/4 (*n* = 5) cats. For proteomic experiment, cortical tissues were collected from control (*n* = 6), CKD1/2 (*n* = 10), and CKD3/4 (*n* = 7) cats, while medullary tissues from control (*n* = 6), CKD1/2 (*n* = 7), and CKD3/4 (*n* = 6) cats. The sample number (*n*) in each group represents individual cats. Bioinformatic and statistical data analyses on metabolomic, RNA-seq and proteomic data were stated in the previous section.

### Diagnosis and IRIS staging of cats

To be eligible for enrollment, cats underwent a complete physical examination, including a 9-point body condition score (BCS)^64^ and muscle condition score (MCS)^65^, and evaluation of medical history, complete blood count (CBC), serum biochemistry profile, SDMA, thyroxine (T4) panel, indirect systolic blood pressure, urinalysis and urinary protein/creatinine ratio. A cat was diagnosed with CKD if urine specific gravity (USG) < 1.035, and serum creatinine or SDMA levels on at least two different assessments, medical history, and physical examination findings supported the diagnosis. Cats diagnosed with CKD were staged according to IRIS guidelines. Cats were staged as IRIS stage 1 based on serum creatinine concentration <1.6 mg/dL, or IRIS stages 2-4 if serum creatinine >1.6 mg/dL. A cat was considered healthy if (1) USG > 1.035, (2) blood SDMA < 18 ug/dL and creatinine <1.6 mg/dL, (3) systolic blood pressure <160 mmHg, and (4) no clinical evidence of kidney disease, urinary tract infection or other major systemic diseases at the time of examination. Diagnosis of cats in cohort 2 was based on renal pathology findings, including assessments on global glomerulosclerosis, fibrosis, inflammation, and tubular atrophy, by a board-certified nephropathologist at a veterinary renal pathology laboratory (Columbus, OH), available health history, as well as clinical observations. Cats were considered for either control or CKD if their renal pathology findings supported the diagnosis. Cats with cancer, heart failure, hyperthyroidism, and diabetes mellitus were excluded. Cats were examined by board-certified veterinarians, staged by a board-certified veterinary internist (S.S.).

### Reporting summary

Further information on research design is available in the Nature Portfolio Reporting Summary linked to this article.

## Supplementary information

Supplementary Information

Description of Additional Supplementary Files

Supplementary Data 1-6

Reporting Summary

## Data Availability

The serum untargeted metabolomics data, renal tissue RNA expression, and protein expression data are provided as Supplementary Data 2–6. The raw sequencing and MS data are also available at the GEO and ProteomeXchange databases with the accession numbers GSE303653 and PXD066590, respectively.
