# Supplementary material for: Integrated multi-omics analysis of renal metabolism in domestic cats with spontaneous chronic kidney disease
Source: Commun Biol. 2025 Dec 13;8:1794. doi: 10.1038/s42003-025-09164-8 (PMC12717052; doi:10.1038/s42003-025-09164-8)
Supplement: Supplementary file 1 — Supplementary Information [file 42003_2025_9164_MOESM1_ESM.pdf]

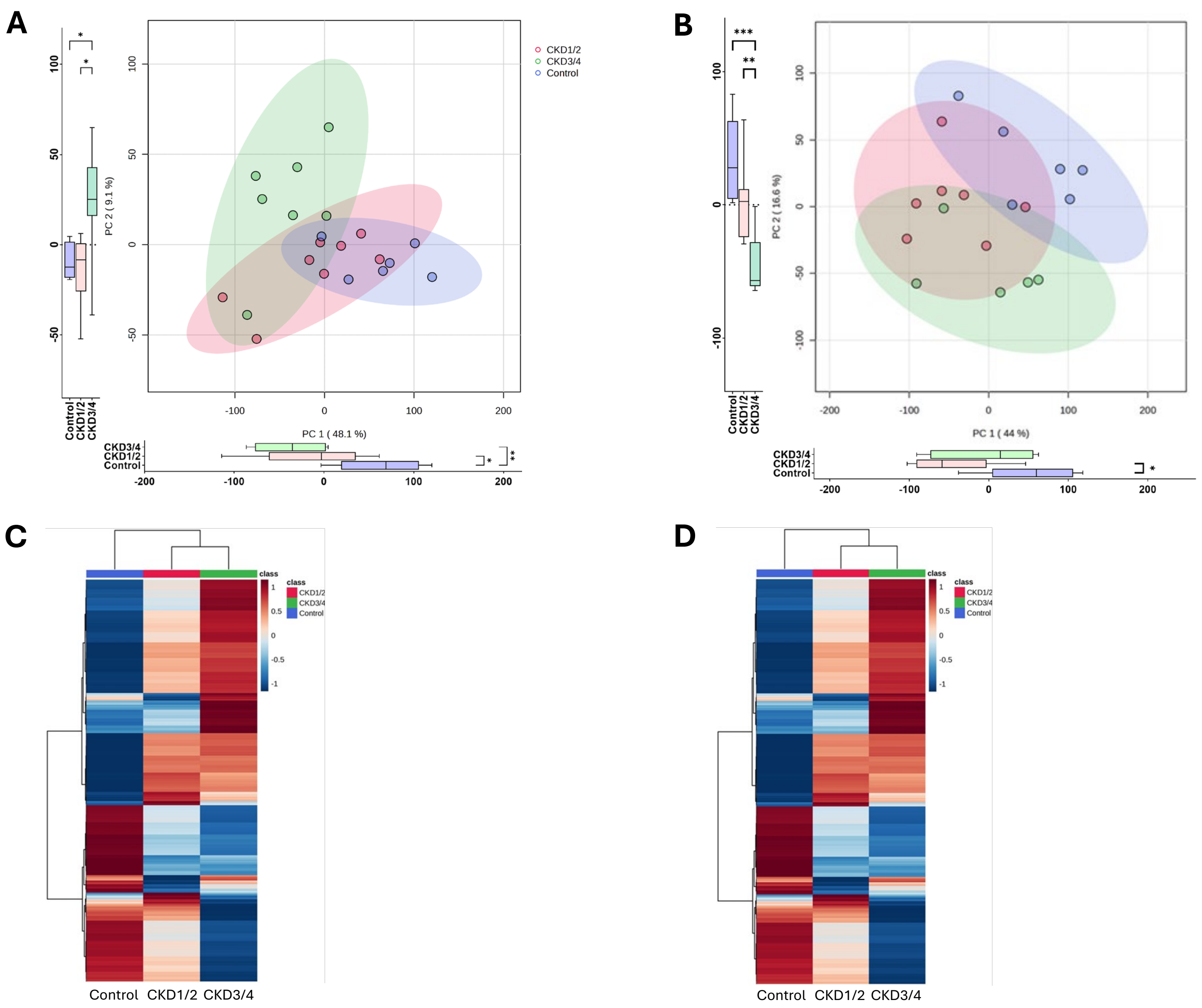

Supplementary Figure 1. Transcriptome-wide changes in response to CKD. The PCA plots of global gene expression in (**A**) the cortex and (**B**) the medulla in the three groups. The heat maps of the average gene expression in (**C**) the cortex and (**D**) the medulla of the three groups. Blue, control; red, CKD1/2; green, CKD3/4. The boxplots below the PCA plots show comparison in PC1 between the three groups. The whiskers extend to 1.5 times the interquartile range from the box. *P<0.05; **P<0.01; ***P<0.001.

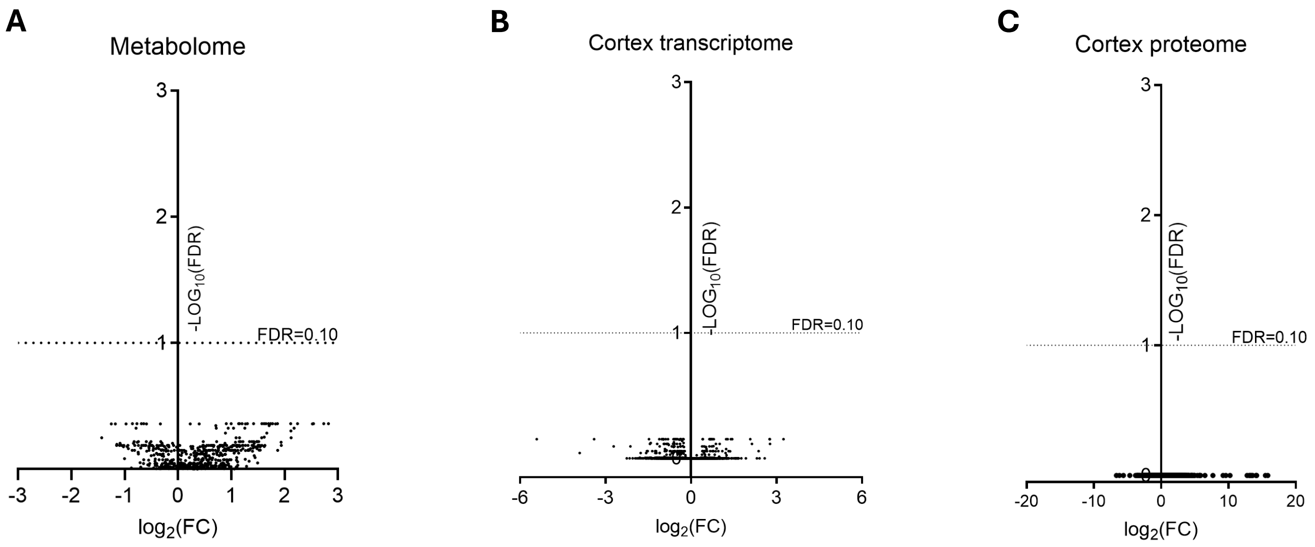

Supplementary Figure 2. Sex effect. Analysis of sex effect in (**A**) metabolome, (**B**) transcriptome of the cortex, and (**C**) proteome in the cortex.

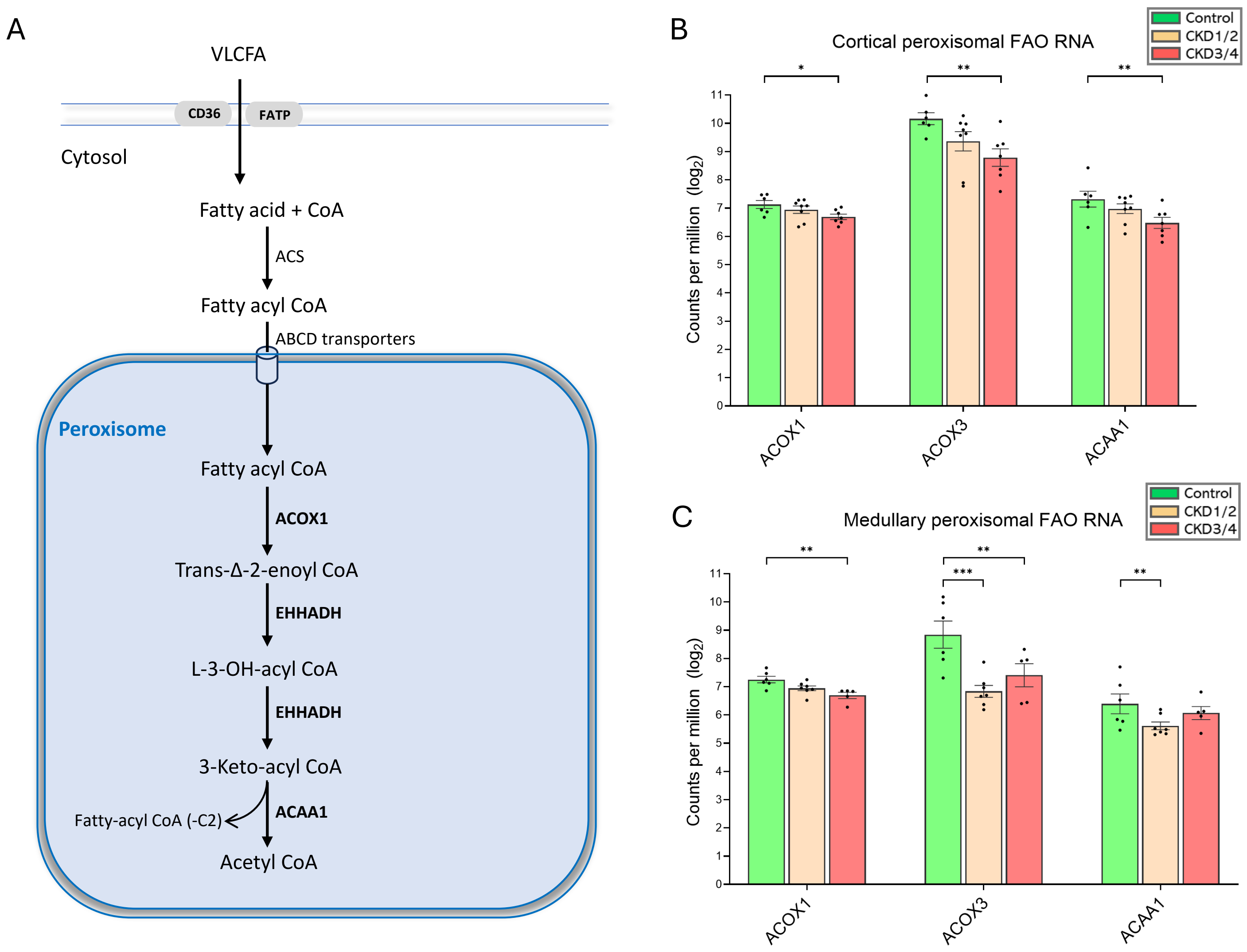

Supplementary Figure 3. Peroxisomal VLCFA β-oxidation. (**A**) Schematic of VLCFA β-oxidation in the peroxisomes. (**B,C**) Expression of genes coding for enzymes involved in peroxisomal VLCFA oxidation in the cortex and medulla, respectively. Sample size for RNA-seq experiments in the cortex (**B**): control (n=6), CKD1/2 (n=8), CKD3/4 (n=7); in the medulla (**C**): control (n=6), CKD1/2 (n=7), CKD3/4 (n=5). Error bars represent the SEM. *P<0.05; **P<0.01; ***P<0.001. **VLCFA**: very long-chain fatty acid, **ACOX1/3**: acyl-CoA oxidase 1 and 3, **ACAA1**: acetyl-CoA acyltransferase 1, **CD36**: FA translocase, **FABP**: FA binding protein, **ACS**: acyl-CoA synthetase, **ABCD**: ATP binding cassette subfamily D, **EHHADH**: enoyl-CoA hydratase and 3-hydroxyacyl CoA dehydrogenase.

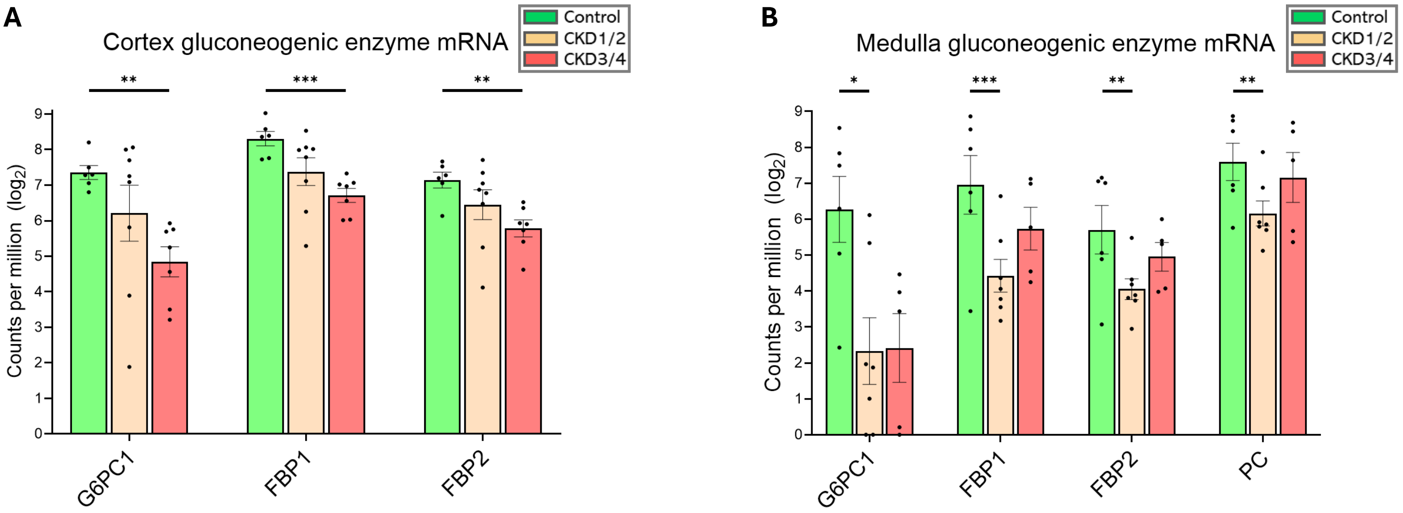

Supplementary Figure 4. (**A**,**B**) Expression of genes coding for enzymes involved in gluconeogenesis in the cortex and medulla, respectively. Sample size for RNA-seq experiments in the cortex (**A**): control (n=6), CKD1/2 (n=8), CKD3/4 (n=7); in the medulla (**B**): control (n=6), CKD1/2 (n=7), CKD3/4 (n=5). Error bars represent the SEM. *P<0.05; **P<0.01; ***P<0.001. **G6PC1**: glucose-6-phosphatase catalytic subunit 1, **FBP1/2**: fructose-bisphosphatase 1 and 2, **PC**: pyruvate carboxylase.

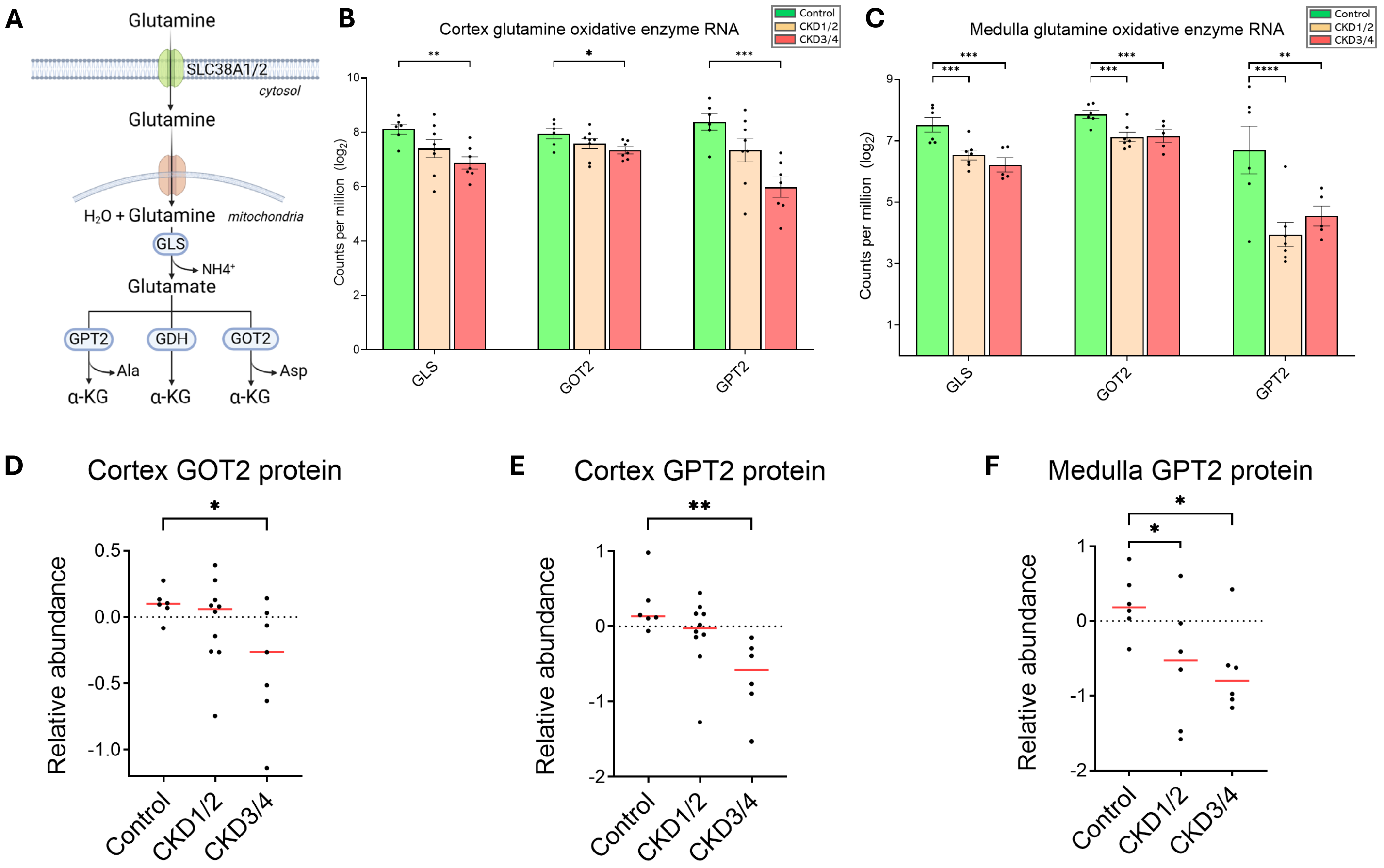

Supplementary Figure 5. (**A**) Schematic of glutamine metabolism. (**B,C**) Expression of genes encoding the enzymes involved in glutamine metabolism in the cortex and medulla, respectively. (**D,E**) Protein expression of mitochondrial GOT2 and GPT2 enzyme proteins in the cortex, respectively. (**F**) Protein expression of mitochondrial GPT2 enzyme protein in the medulla. Sample size for RNA-seq experiments in the cortex (**B**): control (n=6), CKD1/2 (n=8), CKD3/4 (n=7); in the medulla (**C**): control (n=6), CKD1/2 (n=7), CKD3/4 (n=5); Sample size for proteomics experiments in the cortex (**D,E**): control (n=6), CKD1/2 (n=10), and CKD3/4 (n=7) and in the medulla (**F**): control (n=6), CKD1/2 (n=7), and CKD3/4 (n=6). Error bars represent the SEM. Red bars represent the median. *P<0.05; **P<0.01; ***P<0.001; ****P<0.0001. **GLS**: glutaminase, **GOT2**: glutamic-oxaloacetic transaminase 2, **GPT2**: glutamic-pyruvic transaminase 2, **GDH**: glutamate dehydrogenase 1, **α-KG**: alpha-ketoglutarate, **Ala**: alanine, **Asp**: aspartic acid, **SLC38A1/2**: solute carrier family 38 member 1 and 2.

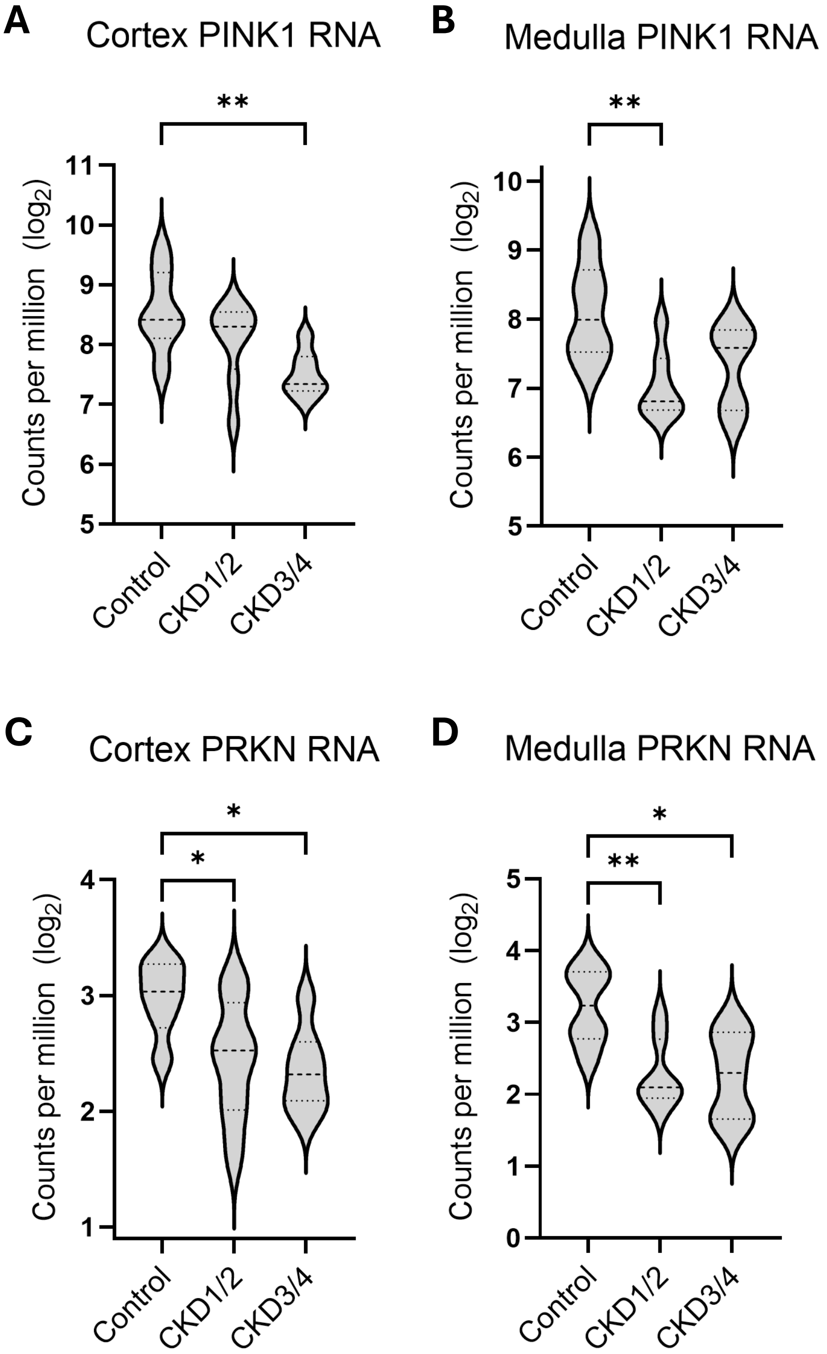

Supplementary Figure 6. Pink1/Parkin-mediated mitophagy pathway. (**A,B**) Expression of PINK1 RNA in the cortex and medulla, respectively, (**C,D**) Expression of PRKN (Parkin) RNA in the cortex and medulla, respectively. Sample size for RNA-seq experiments in the cortex (**A,C**): control (n=6), CKD1/2 (n=8), CKD3/4 (n=7); in the medulla (**B,D**): control (n=6), CKD1/2 (n=7), CKD3/4 (n=5). *P<0.05; **P<0.01. **PINK1**: PTEN induced kinase 1, **PRKN**: parkin RBR E3 ubiquitin protein ligase**.**

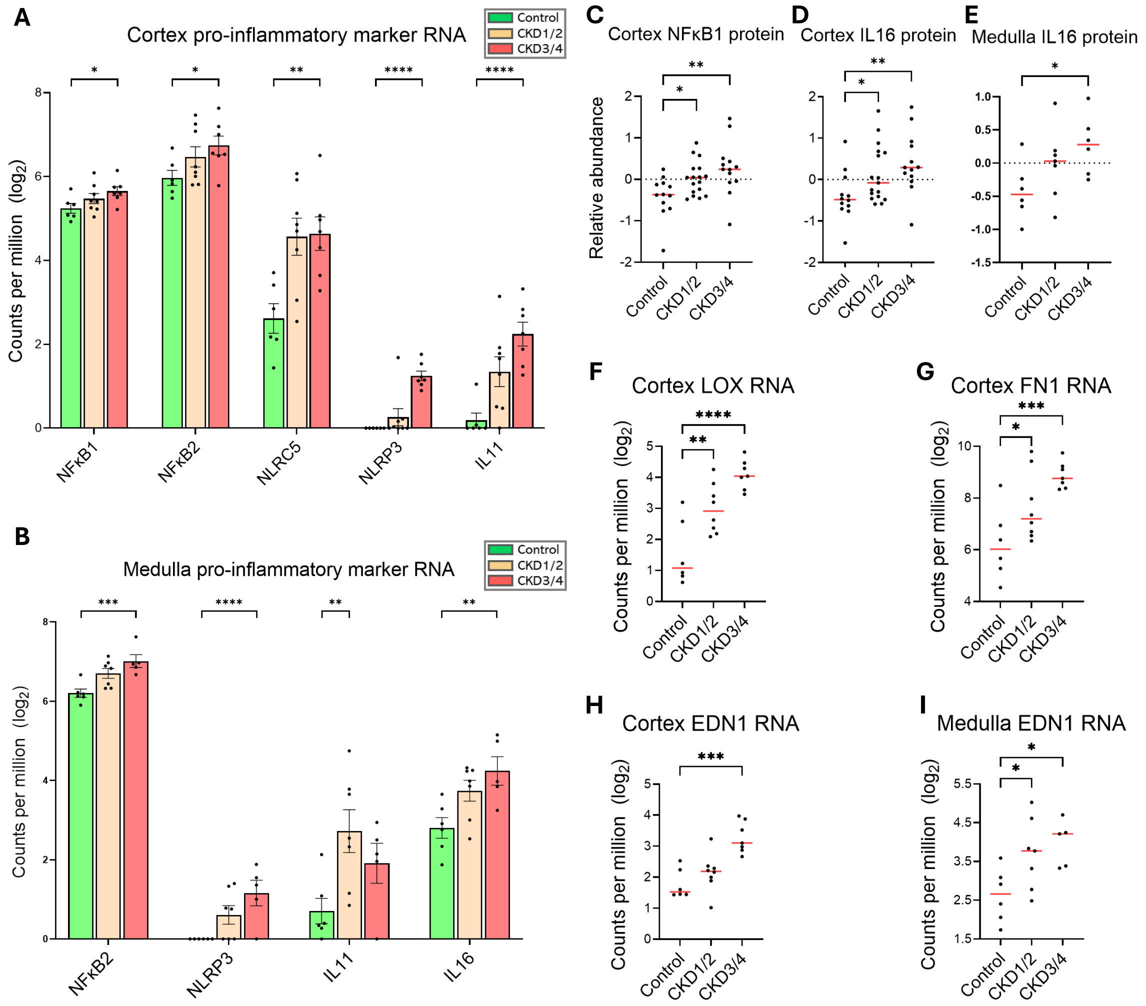

Supplementary Figure 7. Proinflammatory and profibrotic markers. (**A,B**) Gene expression of proinflammatory markers in the cortex and medulla, respectively. (**C,D**) Protein expression of proinflammatory markers NFκB1 and IL16 in the cortex. (**E**) Protein expression of IL16 protein in the medulla. (**F-H**) Gene expression of profibrotic markers LOX, FN1, and EDN1 in the cortex. (**I**) Expression of EDN1 RNA in the medulla. Sample size for RNA-seq experiments in the cortex (**A,F-H**): control (n=6), CKD1/2 (n=8), CKD3/4 (n=7); in the medulla (**B,I**): control (n=6), CKD1/2 (n=7), CKD3/4 (n=5); Sample size for proteomics experiments in the cortex (**C,D**): control (n=6), CKD1/2 (n=10), and CKD3/4 (n=7) and in the medulla (**E**): control (n=6), CKD1/2 (n=7), and CKD3/4 (n=6). Red bars represent the medians. Error bars represent the SEM. *P<0.05; **P<0.01; ***P<0.001; ****P<0.0001. **NFκB1/2**: nuclear factor kappa B subunit 1 and 2, **NLRC5**: NLR family CARD domain containing 5, **NLRP3**: NLR family pyrin domain containing 3, **IL11**: interleukin 11, **IL16**: interleukin 16, **LOX**: lysyl oxidase, **FN1**: fibronectin 1, **EDN1**: endothelin 1.

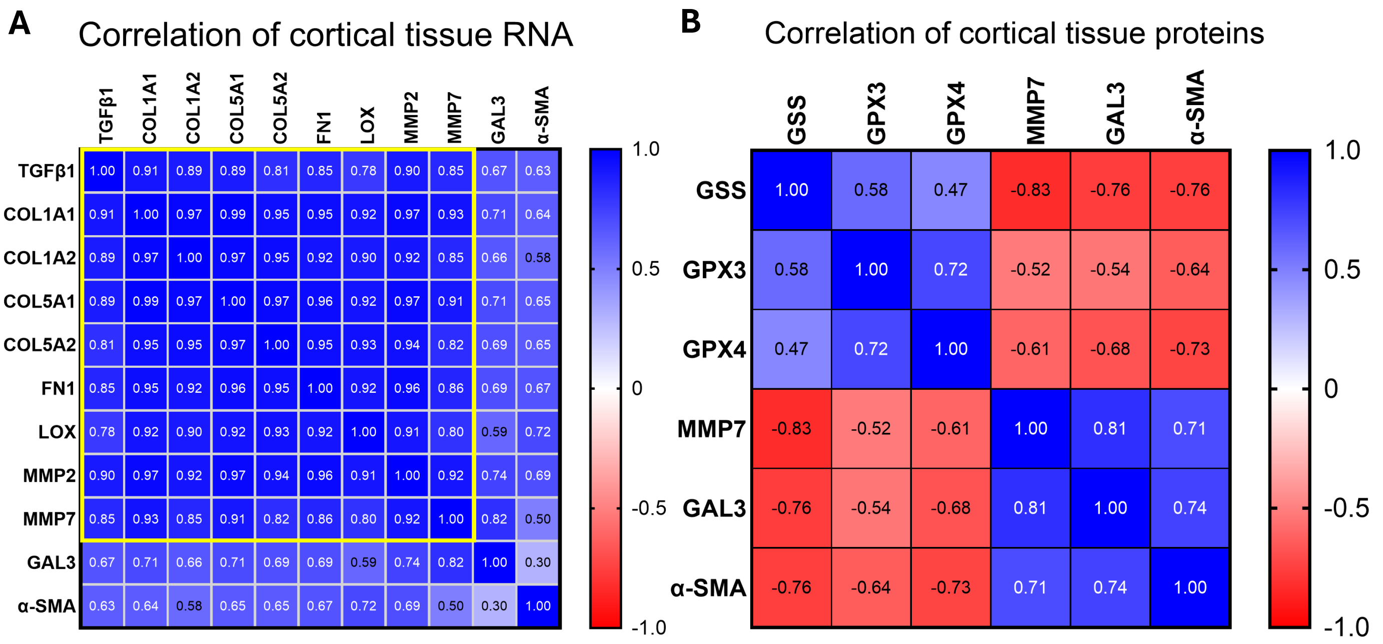

Supplementary Figure 8. Heatmap of Pearson's correlation coefficients (**A**) of cortical tissue RNAs, (**B**) cortical tissue proteins. between redox proteins (GSS, GPX3, and GPX4), and proinflammatory and profibrotic markers (MMP7, GAL3, and α-SMA). Blue and red colors indicate positive and negative correlations, respectively. The numbers in the heatmap denote Pearson's correlation coefficients. **GSS**: glutathione synthetase, **GPX3/4**: glutathione peroxidase 3 and 4, **MMP2/7**: matrix metallopeptidase 2 and 7, **GAL3**: galectin 3, **α-SMA**, alpha smooth muscle actin, **TGFβ1**: transforming growth factor beta 1, **COL1A1/2**: collagen type I α and β chains, **COL5A1/2**: collagen type V α and β chains, **LOX**: lysyl oxidase, **FN1**: fibronectin 1.

Supplementary Table 1. The cohort 1 cats for untargeted serum metabolomics study.

Group	Control	CKD1/2	CKD3/4	P value
Sample size	14	15	6	n/a
**Physical characteristics**				
Age (years)	10.9 ± 0.2	14.9 ± 0.2	11.7 ± 0.3	0.0016
Sex (F/M)	9/5	8/7	5/1	0.48
Weight (kilograms)	4.7 ± 0.1	3.8 ± 0.1	2.9 ± 0.1	0.0007
**Clinical measurements**				
Serum creatinine (mg/dL)	1.44 ± 0.02	1.70 ± 0.03	3.80 ± 0.20	<0.0001
Serum SDMA (ug/dL)	11.0 ± 0.2	16.8 ± 0.3	30.8 ± 1.1	<0.0001
Urine specific gravity	1.0496 ± 0.0004	1.0163 ± 0.0006	1.0105 ± 0.0005	<0.0001
Urine protein/creatinine	0.33 ± 0.05	0.33 ± 0.02	2.35 ± 0.51	0.0015
Proteinuria (N/B/Y)	10/3/1	6/2/7	1/0/4	0.028

[1]Data is presented as mean ± SEM

[2]IRIS guidelines for proteinuria: non-proteinuric (N), UPC<0.2; borderline proteinuric (B), UPC 0.2-0.4 inclusive; proteinuric (Y), UPC>0.4

[3]One cat in the CKD3/4 group had no UPC data

Supplementary Table 2. The cohort 2 cats for renal tissue proteomics and RNA-seq studies.

	RNA-seq								Proteomics							
	Cortex				Medulla				Cortex				Medulla			
	Control	CKD1/2	CKD3/4	P	Control	CKD1/2	CKD3/4	P	Control	CKD1/2	CKD3/4	P	Control	CKD1/2	CKD3/4	P
Size (n)	6	8	7	n/a	6	7	5	n/a	6	10	7	n/a	6	7	6	n/a
Age (y)	13.7	14	12.8	0.9	13.7	13.6	12.2	0.89	13.7	14.3	12.8	0.85	13.7	13.6	13.3	0.99
Breed (S/L)	5/1	7/1	5/2	0.8	5/1	6/1	4/1	1.00	5/1	9/1	5/2	0.79	5/1	6/1	4/2	0.81
Sex (F/M)	4/2	4/4	4/3	0.87	4/2	3/4	3/2	0.84	4/2	6/4	4/3	1.00	4/2	3/4	3/3	0.85

[1]S, domestic short-hair; L, domestic long-hair

[2]P-value from ANOVA test for Age, from Fisher's exact test for Breed and Sex

Supplementary Method 1. Metabolomics

Sample Accessioning: Following receipt, samples were inventoried and immediately stored at -80ºC. Each sample received was accessioned into the Metabolon LIMS system and was assigned by the LIMS a unique identifier that was associated with the original source identifier only. This identifier was used to track all sample handling, tasks, results, etc. The samples (and all derived aliquots) were tracked by the LIMS system. All portions of any sample were automatically assigned their own unique identifiers by the LIMS when a new task was created; the relationship of these samples was also tracked. All samples were maintained at -80ºC until processed.

Sample Preparation: Samples were prepared using the automated MicroLab STAR® system from Hamilton Company. Several recovery standards were added prior to the first step in the extraction process for QC purposes. To remove protein, dissociate small molecules bound to protein or trapped in the precipitated protein matrix, and to recover chemically diverse metabolites, proteins were precipitated with methanol under vigorous shaking for 2 min (Glen Mills GenoGrinder 2000) followed by centrifugation. The resulting extract was divided into five fractions: two for analysis by two separate reverse phase (RP)/UPLC-MS/MS methods with positive ion mode electrospray ionization (ESI), one for analysis by RP/UPLC-MS/MS with negative ion mode ESI, one for analysis by HILIC/UPLC-MS/MS with negative ion mode ESI, and one sample was reserved for backup. Samples were placed briefly on a TurboVap® (Zymark) to remove the organic solvent. The sample extracts were stored overnight under nitrogen before preparation for analysis.

QA/QC: Several types of controls were analyzed in concert with the experimental samples: a pooled matrix sample generated by taking a small volume of each experimental sample (or alternatively, use of a pool of well-characterized human plasma) served as a technical replicate throughout the data set; extracted water samples served as process blanks; and a cocktail of QC standards that were carefully chosen not to interfere with the measurement of endogenous compounds were spiked into every analyzed sample, allowed instrument performance monitoring and aided chromatographic alignment. **Illustrations 1 and 2** describe these QC samples and standards. Instrument variability was determined by calculating the median relative standard deviation (RSD) for the standards that were added to each sample prior to injection into the mass spectrometers. Overall process variability was determined by calculating the median RSD for all endogenous metabolites (i.e., non-instrument standards) present in 100% of the pooled matrix samples. Experimental samples were randomized across the platform run with QC samples spaced evenly among the injections, as outlined in **Illustration 3**.

Illustration 1. Description of Metabolon QC Samples

Type	Description	Purpose
MTRX	Large pool of human plasma maintained by Metabolon that has been characterized extensively.	Assure that all aspects of the Metabolon process are operating within specifications.
CMTRX	Pool created by taking a small aliquot from every customer sample.	Assess the effect of a non-plasma matrix on the Metabolon process and distinguish biological variability from process variability.
PRCS	Aliquot of ultra-pure water	Process Blank used to assess the contribution to compound signals from the process.
SOLV	Aliquot of solvents used in extraction.	Solvent Blank used to segregate contamination sources in the extraction.

Illustration 2. Metabolon QC Standards

Type	Description	Purpose
RS	Recovery Standard	Assess variability and verify performance of extraction and instrumentation.
IS	Internal Standard	Assess variability and performance of instrument.

Illustration 3. Preparation of client-specific technical replicates. A small aliquot of each client sample (colored cylinders) is pooled to create a CMTRX technical replicate sample (multi-colored cylinder), which is then injected periodically throughout the platform run. Variability among consistently detected biochemicals can be used to calculate an estimate of overall process and platform variability.

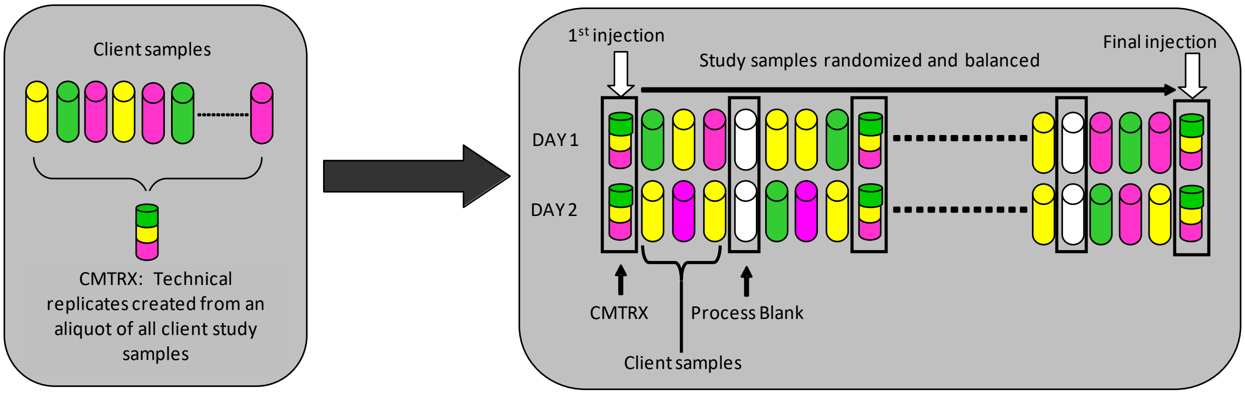

Ultrahigh Performance Liquid Chromatography-Tandem Mass Spectroscopy (UPLC-MS/MS): All methods utilized a Waters ACQUITY ultra-performance liquid chromatography (UPLC) and a Thermo Scientific Q-Exactive high resolution/accurate mass spectrometer interfaced with a heated electrospray ionization (HESI-II) source and Orbitrap mass analyzer operated at 35,000 mass resolution. The sample extract was dried then reconstituted in solvents compatible to each of the four methods. Each reconstitution solvent contained a series of standards at fixed concentrations to ensure injection and chromatographic consistency. One aliquot was analyzed using acidic positive ion conditions, chromatographically optimized for more hydrophilic compounds. In this method, the extract was gradient eluted from a C18 column (Waters UPLC BEH C18-2.1x100 mm, 1.7 µm) using water and methanol, containing 0.05% perfluoropentanoic acid (PFPA) and 0.1% formic acid (FA). Another aliquot was also analyzed using acidic positive ion conditions, however it was chromatographically optimized for more hydrophobic compounds. In this method, the extract was gradient eluted from the same afore mentioned C18 column using methanol, acetonitrile, water, 0.05% PFPA and 0.01% FA and was operated at an overall higher organic content. Another aliquot was analyzed using basic negative ion optimized conditions using a separate dedicated C18 column. The basic extracts were gradient eluted from the column using methanol and water, however with 6.5mM Ammonium Bicarbonate at pH 8. The fourth aliquot was analyzed via negative ionization following elution from a HILIC column (Waters UPLC BEH Amide 2.1x150 mm, 1.7 µm) using a gradient consisting of water and acetonitrile with 10mM Ammonium Formate, pH 10.8. The MS analysis alternated between MS and data-dependent MS^n scans using dynamic exclusion. The scan range varied slighted between methods but covered 70-1000 m/z. Raw data files are archived and extracted as described below.

Bioinformatics: The informatics system consisted of four major components, the Laboratory Information Management System (LIMS), the data extraction and peak-identification software, data processing tools for QC and compound identification, and a collection of information interpretation and visualization tools for use by data analysts. The hardware and software foundations for these informatics components were the LAN backbone, and a database server running Oracle 10.2.0.1 Enterprise Edition.

LIMS: The purpose of the Metabolon LIMS system was to enable fully auditable laboratory automation through a secure, easy to use, and highly specialized system. The scope of the Metabolon LIMS system encompasses sample accessioning, sample preparation and instrumental analysis and reporting and advanced data analysis. All of the subsequent software systems are grounded in the LIMS data structures. It has been modified to leverage and interface with the in-house information extraction and data visualization systems, as well as third party instrumentation and data analysis software.

Data Extraction and Compound Identification: Raw data was extracted, peak-identified and QC processed using Metabolon's hardware and software. These systems are built on a web-service platform utilizing Microsoft's .NET technologies, which run on high-performance application servers and fiber-channel storage arrays in clusters to provide active failover and load-balancing. Compounds were identified by comparison to library entries of purified standards or recurrent unknown entities. Metabolon maintains a library based on authenticated standards that contains the retention time/index (RI), mass to charge ratio (*m/z)*, and chromatographic data (including MS/MS spectral data) on all molecules present in the library. Furthermore, biochemical identifications are based on three criteria: retention index within a narrow RI window of the proposed identification, accurate mass match to the library +/- 10 ppm, and the MS/MS forward and

reverse scores between the experimental data and authentic standards. The MS/MS scores are based on a comparison of the ions present in the experimental spectrum to the ions present in the library spectrum. While there may be similarities between these molecules based on one of these factors, the use of all three data points can be utilized to distinguish and differentiate biochemicals. More than 3300 commercially available purified standard compounds have been acquired and registered into LIMS for analysis on all platforms for determination of their analytical characteristics. Additional mass spectral entries have been created for structurally unnamed biochemicals, which have been identified by virtue of their recurrent nature (both chromatographic and mass spectral). These compounds have the potential to be identified by future acquisition of a matching purified standard or by classical structural analysis.

Curation: A variety of curation procedures were carried out to ensure that a high quality data set was made available for statistical analysis and data interpretation. The QC and curation processes were designed to ensure accurate and consistent identification of true chemical entities, and to remove those representing system artifacts, mis-assignments, and background noise. Metabolon data analysts use proprietary visualization and interpretation software to confirm the consistency of peak identification among the various samples. Library matches for each compound were checked for each sample and corrected if necessary. Metabolite Quantification and Data Normalization: Peaks were quantified using area-under-the-curve. For studies spanning multiple days, a data normalization step was performed to correct variation resulting from instrument inter-day tuning differences. Essentially, each compound was corrected in run-day blocks by registering the medians to equal one (1.00) and normalizing each data point proportionately (termed the "block correction"; **Illustration 4**). For studies that did not require more than one day of analysis, no normalization is necessary, other than for purposes of data visualization. In certain instances, biochemical data may have been normalized to an additional factor (e.g., cell counts, total protein as determined by Bradford assay, osmolality, etc.) to account for differences in metabolite levels due to differences in the amount of material present in each sample.

Illustration 4. Visualization of data normalization steps for a multiday platform run.

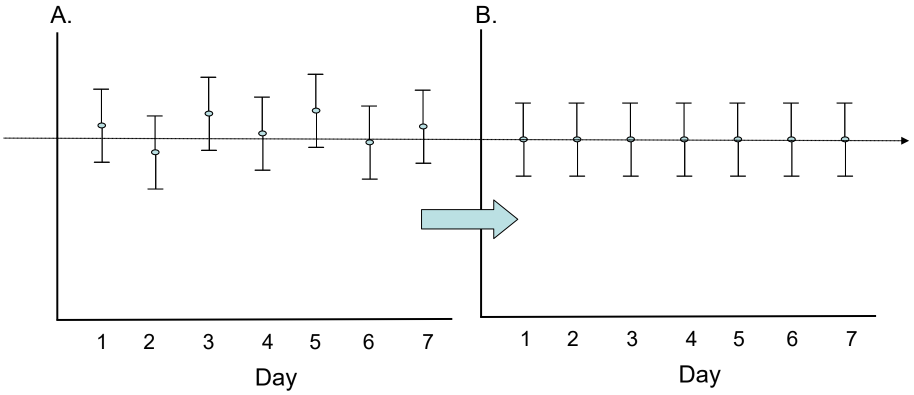

Supplementary Method 2. RNA sequencing

Total RNA was extracted using the Agencourt RNAdvance Tissue Kit (Beckman Coulter): tissues were disrupted and lyzed using the Fastprep 2 x 1' at speed 6. 400 µL of lyzate was extracted and eluted in 50µL. The RNA was quantified using Quant It Ribogreen assay (Life Technologies) and its quality was checked on a Fragment Analyzer (Agilent). The samples with an RQN above 4.0 are considered to have passed the quality check. The Stranded mRNA Library Prep protocol convert the mRNA in a total RNA sample into a library of template molecules of known strand origin using the reagents provided in an Illumina® Stranded mRNA library prep workflow. 500ng of total RNA was used of each RNA sample. A bead-based mRNA capture followed by a fragmentation, first and second strand synthesis, an end repair and a 3' adenylation and indexed linker ligation was done. The adapter ligated cDNA library fragments were than enriched by PCR using 13 cycles of amplification. The library size was controlled with the High Sensitivity NGS Fragment Analysis kit on a Fragment Analyzer. The libraries were quantified with Qubit dsDNA HS Assay Kits (Thermo Fisher Scientific). The sequencing was performed on NextSeq 2000 with V3 chemistry PE 150 cycles (Illumina), loaded at 750pM and with 1% PhiX. Six V3 300 flow cells were used to achieve the sequencing depth needed per sample (~100 million reads). Primary data QC was performed during the sequencing run to ensure the optimal flow cell loading (cluster density) and check the quality metrics of the sequencing run (QC 30). The percentage ≥Q30, means the percentage of bases with a quality Phred score of 30 or higher. It is a measure of the quality of the identification of the nucleobases generated. The Phred score is logarithmically linked to error probabilities. A Phred score equal to 30 means a probability of incorrect base assignment of 1 in 1,000, which corresponds to a base call accuracy of 99.9%. Regarding Illumina specification, the score should be a minimum of 80%. All six flow-cells met Illumina requirements. Sequencing raw data were demultiplexed and transformed into fastq files using casava v1.8.2.

The fastq sequencing files were aligned against the domestic cat reference genomes Felis_catus_9.0 using RNAstar v2.5.3. The RNAstar options --twopassMode Basic --sjdbOverhang 75 --outSAMtype BAM SortedByCoordinate were used. The output bam files were merged using samtools v1.4. The bam files were then filtered to include only sequences which mapped once and where the number of mismatches does not exceed 5. The counts per gene were generated using htseq_count v2.16.2. The raw counts were converted to counts per million by the cpm function from the R package "edgeR" (Robinson, McCarthy, & Smyth, 2010). Genes with less than 2 counts per million in at least 5 samples were discarded. The remaining sequencing data were normalized by the trimmed mean of M-values (TMM) method as implemented in the function calcNormFactors in edgeR. Differentially expressed genes were defined by fitting a quasi-likelihood negative binomial generalized log-linear model to count data using the glmQLFTest function also implemented in edgeR. The Molecular Signature Database (MSigDB; Subramanian, Tamayo, et al. 2005, PNAS) was used to perform pathway analysis across Biocarta, KEGG, Reactome and Gene Ontology.

Supplementary Method 3. Proteomics

An amount of 20-30 mg of sample tissue was weighed into a Lysing matrix D tube (MP Biomedicals). Samples were extracted in 20 µL RIPA lysis buffer (Sigma-Aldrich) per mg of tissue, in the presence of protease inhibitors (cOmplete™, Mini, EDTA-free Protease Inhibitor Cocktail from Roche Diagnostics), and using the Fast Prep 24 apparatus (MP Biomedicals) for 40 s at 6 m/s (five lysis cycles were performed). Supernatants were recovered after centrifugation at 4 °C and then frozen at -20 °C. Protein concentration in the extracts was determined with the Pierce BCA protein assay (Thermo Scientific). Sample pools combining individual samples were prepared. For each individual sample or sample pools, a sample volume corresponding to 100 µg of proteins was spiked with 45 µL of a β-lactoglobulin (LACB, Sigma-Aldrich) solution at 0.014 µg/ µL in triethylammonium hydrogen carbonate buffer (TEAB, Sigma-Aldrich) 200 mM. Sample volume was then adjusted to 100 µL with H2O. Proteins were reduced by adding 10 µL of tris (2-carboxyethyl) phosphine hydrochloride (Sigma-Aldrich) 200 mM in H2O, and then alkylated with 10 µL iodoacetamide (Sigma-Aldrich) 370 mM in TEAB. Denatured proteins were precipitated with 600 µL of cold acetone (Sigma-Aldrich) for 1 hour at -20 °C, and the recovered pellets were evaporated to dryness using a SpeedVac concentrator (Thermo Scientific). The protein pellets were then resuspended in 100 µL TEAB buffer 100 mM. A volume of 10 µL of 0.25 µg/µL trypsin/Lys-C mix (Promega) was added, and digestion was performed overnight at 37 °C. Samples and sample pools were individually labeled using tandem mass tag (TMT) 6-plex (Thermo Scientific) following the manufacturer's instructions. Reaction was stopped by adding 8 µL of hydroxylamine (Sigma-Aldrich) 5%. Samples were then pooled into different TMT experiments. Each resulting TMT pooled sample went through reversed-phase (RP) followed by strong cation-exchange purifications as previously described. The purified peptides were evaporated to dryness using a SpeedVac centrifuge. Dried peptides were resuspended into 1.5 mL of H2O/CH3CN/FA (97/3/0.1) to reach a theoretical peptide concentration of 0.4 µg/µL.

Volumes of 4 µL were injected and samples were analyzed in triplicate by RP-liquid chromatography (LC)-tandem MS (MS/MS) using an Ultimate 3000 RSLC Nano coupled with an Orbitrap Fusion LUMOS (Thermo Scientific). Peptides were first trapped on an Acclaim PepMap 300 µm × 5 mm (C18, 5 µm, 100 Å) pre-column (Thermo Scientific) and then eluted on an Acclaim PepMap RSLC 75 µm × 50 cm (C18, 2 µm, 100 Å) nano column (Thermo Scientific) heated at 50 °C at a flow rate of 0.3 µL/min, starting with 6.3% of mobile phase B (H2O/CH3CN/FA (20/80/0.08)) and 93.7% of mobile phase A (H2O/CH3CN/FA (98/2/0.1)), and reaching 40% of mobile phase B after 157 min. MS data were acquired using a data-dependent method. Ion spray voltage of 1900 V and a transfer tube temperature of 275 °C were set up. For MS survey scans in profile mode, the Orbitrap resolution was 120000 at m/z = 200 (automatic gain control (AGC) target of 2 × 105) with a m/z scan range 300-1500, and a maximum injection time of 100 ms. For MS/MS with higher-energy collisional dissociation at 35% of the normalized collision energy, AGC target was set to 1 × 105 (isolation width of 0.7 in the quadrupole), with a resolution of 30000 at m/z = 200, first mass at m/z = 100, and a maximum injection time of 105 ms with Orbitrap acquiring in profile mode. A duty cycle time of 3 s (top speed mode) was chosen. Ions were injected for all available parallelizable time. Dynamic exclusion was set for 60 s within a ± 10 ppm window.

Protein identifications were obtained using Mascot 2.6.1 through the Mascot Daemon (Matrix Science,). Searches of MS raw files were done against the UniProt Felis catus reference proteome database (40213 protein entries, version 2020-11-18) including LACB sequence using the following parameters: Enzyme: Trypsin; Fixed modifications:

Carbamidomethyl (C), TMT6plex (K); Variable modifications: Deamidated (NQ), Oxidation (M), TMT6plex (N-term); Peptide mass tolerance: ± 10 ppm; Fragment mass tolerance: ± 0.02 Da; Max missed cleavages: 2. Qualitative and quantitative data analysis was performed with Scaffold Q+ version 4.8.8 (Proteome Software,). A further database search was done with X! Tandem (version Alanine (2017.2.1.4)). Protein and peptide FDR thresholds were set at 1%. Protein identifications were accepted if they contained at least two identified peptides. The triplicate quantitative values were average for each protein and sample. Each sample was compared to the pooled reference from all samples in each tissue type. Proteins with more than 70% of missing values were removed from the analysis, resulting in 2774 medullar proteins and 3047 cortical proteins. A linear model was fitted to each protein adjusting for sex and compared each CKD groups with control. The p-values associated from the linear models were adjusted for multiple testing error using the Benjamini-Hochberg method.

In many clinical settings, FDR<0.05 has been suggested as being too low to embrace all potential meaningful results[1]. In some studies, less restricted statistical approaches, including P<0.05, were proposed and applied[2, 3]. In the context of multiomics analysis, we applied FDR <0.30 cutoff for differentially expressed proteins (DEPs).
