# Supplementary material for: Integrated multi-omics analysis of renal metabolism in domestic cats with spontaneous chronic kidney disease
Source: Commun Biol. 2025 Dec 13;8:1794. doi: 10.1038/s42003-025-09164-8 (PMC12717052; doi:10.1038/s42003-025-09164-8)
Supplement: Supplementary file 2 — Description of Additional Supplementary Files [file 42003_2025_9164_MOESM2_ESM.pdf]

Description of Additional Supplementary Files

File name: Supplementary Data 1

Description: The list of significant metabolites

File name: Supplementary Data 2

Description: Untargeted serum metabolomics data

File name: Supplementary Data 3

Description: RNA expression data of renal cortex (log2 counts per million)

File name: Supplementary Data 4

Description: RNA expression data of renal medulla (log2 counts per million)

File name: Supplementary Data 5

Description: Protein expression data of the renal cortex.

File name: Supplementary Data 6

Description: Protein expression data of renal medulla.
